# Aromatic vs. Aliphatic Hyperbranched Polyphosphoesters as Flame Retardants in Epoxy Resins

**DOI:** 10.3390/molecules24213901

**Published:** 2019-10-29

**Authors:** Jens C. Markwart, Alexander Battig, Maria M. Velencoso, Dennis Pollok, Bernhard Schartel, Frederik R. Wurm

**Affiliations:** 1 Max-Planck-Institut für Polymerforschung, Ackermannweg 10, 55128 Mainz, Germany; markwart@mpip-mainz.mpg.de (J.C.M.); maria.mvelencoso@gmail.com (M.M.V.); pollok@uni-mainz.de (D.P.); 2 Graduate School Materials Science in Mainz, Staudinger Weg 9, 55128 Mainz, Germany; 3 Bundesanstalt für Materialforschung und-prüfung (BAM), Unter den Eichen 87, 12205 Berlin, Germany; alexander.battig@bam.de

**Keywords:** phosphorus, metathesis, dendritic, cone calorimeter, fire test

## Abstract

The current trend for future flame retardants (FRs) goes to novel efficient halogen-free materials, due to the ban of several halogenated FRs. Among the most promising alternatives are phosphorus-based FRs, and of those, polymeric materials with complex shape have been recently reported. Herein, we present novel halogen-free aromatic and aliphatic hyperbranched polyphosphoesters (hbPPEs), which were synthesized by olefin metathesis polymerization and investigated them as a FR in epoxy resins. We compare their efficiency (aliphatic vs. aromatic) and further assess the differences between the monomeric compounds and the hbPPEs. The decomposition and vaporizing behavior of a compound is an important factor in its flame-retardant behavior, but also the interaction with the pyrolyzing matrix has a significant influence on the performance. Therefore, the challenge in designing a FR is to optimize the chemical structure and its decomposition pathway to the matrix, with regards to time and temperature. This behavior becomes obvious in this study, and explains the superior gas phase activity of the aliphatic FRs.

## 1. Introduction

The overlap of the decomposition temperatures of a flame retardant (FR) and its polymer matrix is essential for its effectiveness in the case of fire [1,2,3,4]. Aromatic polymers typically have higher thermal stability than aliphatic polymers. In the case of a FR, this higher thermal stability influences at which temperatures the active species are available in the gas phase. An important example of this behavior was reported for brominated aromatic and aliphatic FRs in polypropylene. The thermal decomposition of aliphatic FRs starts below the thermal decomposition of polypropylene, which leads to a good performance in this matrix. Aromatic FRs decompose after polypropylene and therefore, at the decomposition temperature of polypropylene, no optimal debromination is achieved, resulting in mediocre performance [5].

**Scheme 1:**
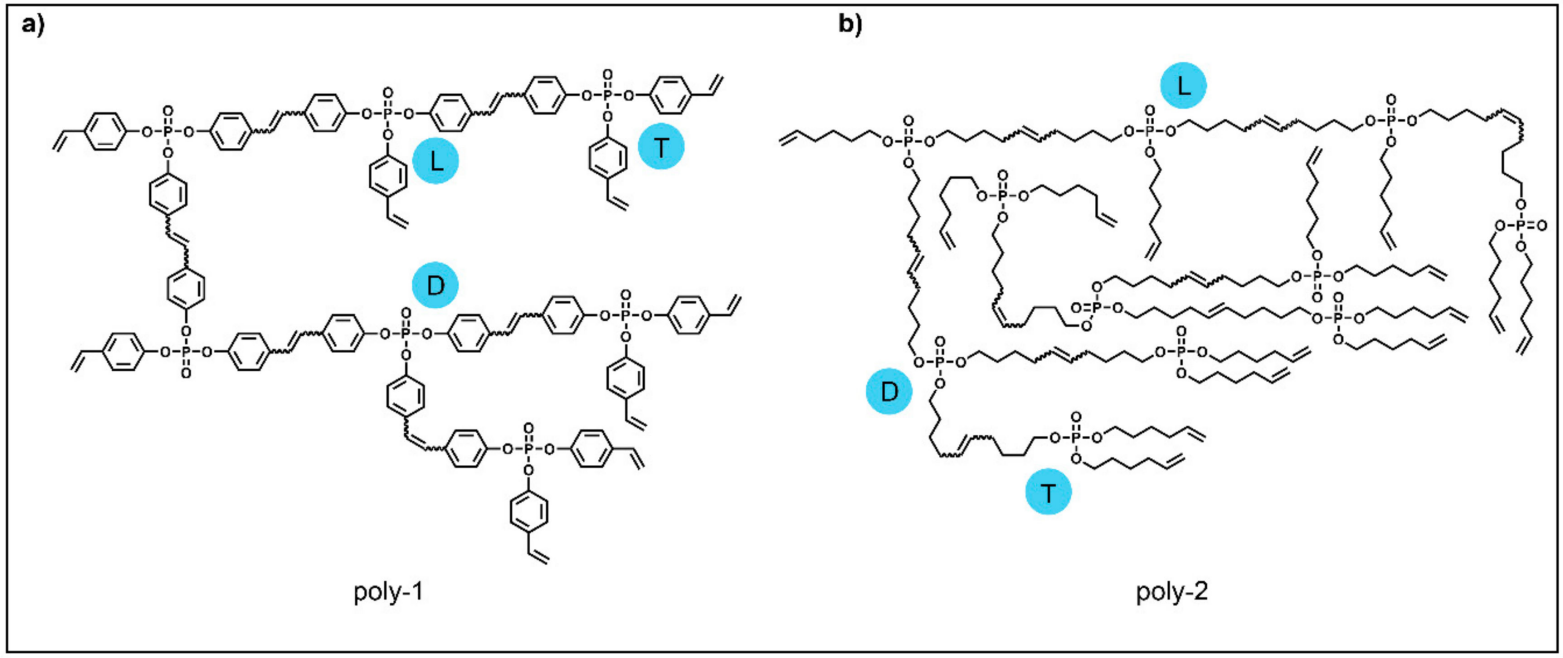
Chemical structures of the investigated polymers: a) the aromatic poly-1 and b) the aliphatic poly-2.

Here, we compared an aromatic with an aliphatic hyperbranched polyphosphoester (hbPPE) prepared via acyclic triene metathesis (ATMET) polymerization as additive FRs in epoxy resins (Scheme 1). We discuss the structure-property relationship and utilize thermal analysis to understand the flame-retardant mechanism and investigate the influence of the higher thermal stability of the aromatic compounds and the resulting availability of phosphorus in the gas phase [5].

In spite of their high flammability, the excellent mechanical and insulating properties of epoxy resins have led to their widespread application as lightweight material products for construction or electrical equipment. In 2016, the market for epoxy resins had a volume of US$ 21.5 billion [6]. Typically, epoxy resins are treated with FRs in many applications.

For several decades, halogenated FRs were used as effective FRs. However, in recent years, due to their potential harm to health and environment, their use has been legislatively restricted around the world, promoting a growing demand for halogen-free alternatives, of which phosphorus is an attractive alternative [7]. Phosphorus in the gas phase is reported to be similar or even superior to hydrogen halides like HBr [8], has attractive plasticizing properties [9], adjustable hydrophilicity [10] and potential degradability [11] and biocompatibility [12].

The versatility of polymer chemistry was used to address problems of low molar mass FRs, such as poor matrix compatibility, leaching or migration out of the polymer matrix. In the literature, polyphosphoesters are gaining increased attention as a promising class of polymeric FRs because of the aforementioned reasons and their versatile chemistry that allows tuning their chemical structure to control degradation behavior and matrix compatibility. Branched polymers with their high number of reactive or end-groups, lower intrinsic viscosities and higher matrix compatibility compared to linear polymers are interesting candidates for flame retardant additives [13,14,15,16]. Also, PPEs can be prepared as branched polymers, typically relying on the pentavalency of phosphorus [17]. However, the majority of hbPPEs reported in literature are aliphatic materials, only a few publications have reported on aromatic hbPPEs [18,19,20,21,22]. The field is dominated mostly by classical linear aromatic phosphates, such as resorcinol bis(diphenyl phosphate) or bisphenol A bis(diphenyl phosphate) due to their good flame retardant performance, but typically with relatively low molar mass and definition [23,24,25]. To further understand the flame retardant mechanism and improve performance, additional research in the field of hbPPEs is necessary and this work contributes to this development. 

## 2. Results

**Figure 1:**
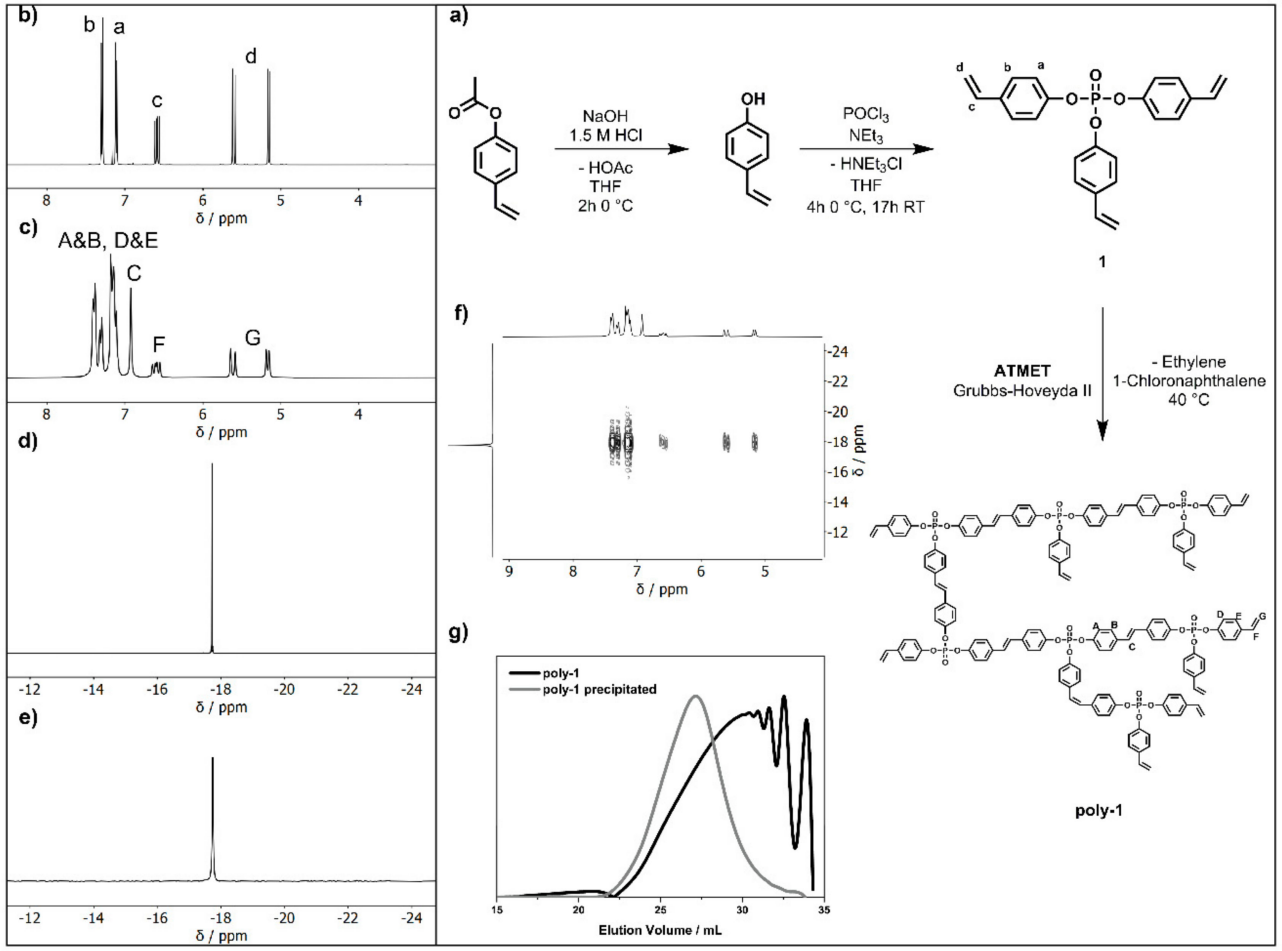
(**a**) Synthesis scheme of tris(p-vinylphenyl)phosphate (1) and poly(tris(p-vinylphenyl)phosphate) (hbPPE); (**b**) ^1^H NMR spectra of 1; (**c**) ^1^H NMR spectra of poly-1; (**d**) ^31^P NMR spectra of 1; (**e**) ^31^P NMR spectra of poly-1; (**f**) ^1^H^31^P HMBC spectra of poly-1; (**g**) SEC curves of poly-1 before and after precipitation in Toluene.

Acyclic diene metathesis polymerization (ADMET) is a versatile technique to prepare a broad range of linear functional polymers [26,27,28,29,30]. Olefin metathesis is also able to polymerize an A_3_ monomer without any complementary B_2_ monomer needed to give a hyperbranched structure [15]. For the synthesis of aromatic hbPPEs via acyclic triene metathesis (ATMET) polymerization, an aromatic phosphoester with vinyl groups is mandatory. In this study, the synthesis of tris(p-vinylphenyl)phosphate (1) was performed in a single reaction step from POCl_3_ and 4-vinylphenol without further purification such as distillation or chromatography, thus obtaining the A_3_-monomer in high purity and yield (Figure 1a). The resulting monomer is a liquid at room temperature and has a phosphorus content of 7.66 wt.-%. 1 is soluble in aromatic solvents (e.g., toluene) and halogenated solvents (e.g., dichloromethane and chloroform), and insoluble in water. 1 proved thermally stable until a temperature of 127 °C (measured by TGA), at which the vinyl groups undergo radical cross-linking, due mainly to the electron-withdrawing resonance effect of the adjacent phosphate at the ring. The cross-linking-reaction was proven by heating 1 to 300 °C in a silicon form for 2 h producing a hard, cross-linked PPE (Figure S23).

1 was used as a novel A_3_ monomer for the ATMET polymerization to produce poly-1. The protocol of the ATMET polymerization of 1 is described in Figure 1 a and in detail in the Experimental Part. The monomer was dissolved together with the respective Grubbs catalyst in a 37 wt.-% solution of 1-chloronaphthalene at 40 °C and the polymerization was conducted for 5 min in vacuo. Poly-1 was used as a flame-retardant additive in epoxy resins after precipitation into cold hexane. No polymerization in the bulk conditions at 60 °C was observed, probably due to the high viscosity of the monomer. We carried out polymerizations with Grubbs 1^st^ generation catalyst at 40 °C and 60 °C, but almost only oligomers were observed (*M*_n_ < 1200 g mol^−1^ from size exclusion chromatography (SEC), Figure S1). When Grubbs Hoveyda 2^nd^ generation catalyst was used, a broad range of molecular weights was obtained, depending on the reaction conditions (Table S1 and Figure 1g). SEC showed a molar mass of ca. *M*_n_ 3000 g mol^−1^ with dispersity *M*_w_/*M*_n_ ≈ 3 after 120 min of reaction. However, when the addition of the catalyst was carried out in two phases, higher apparent molar masses of ca. *M*_n_ 4800 g mol^−1^ with broad molar mass distribution (*M*_w_/*M*_n_ ≈ 11) were obtained. Longer reaction times led to cross-linking of the material. To prevent cross-linking, the reaction can be terminated by the addition of ethyl vinyl ether or methyl acrylate. The resulting polymer was partially soluble in aromatic solvents (e.g., toluene) but proved highly soluble in halogenated solvents (e.g., dichloromethane and chloroform). Extraction of the crude polymer with toluene resulted in the precipitation of the high molecular weight fractions, providing a poly-1 sample with a molar mass of ca. *M*_n_ 11,500 g mol^−1^ and *M*_w_/*M*_n_ = 1.7 from SEC (Figure 1g).

The polymerization of 1 was followed by ^1^H NMR spectroscopy (Figure 1b,c). After polycondensation, the resonances of the terminal double bonds at 5.16 ppm, 5.61 ppm and 6.59 ppm decreased, and a new signal at 6.92 ppm for the internal double bonds was detected. The ^31^P NMR spectrum revealed a distinct signal at the same chemical environment (−17.73 ppm) of the monomer (Figure 1d,e). 

**Figure 2:**
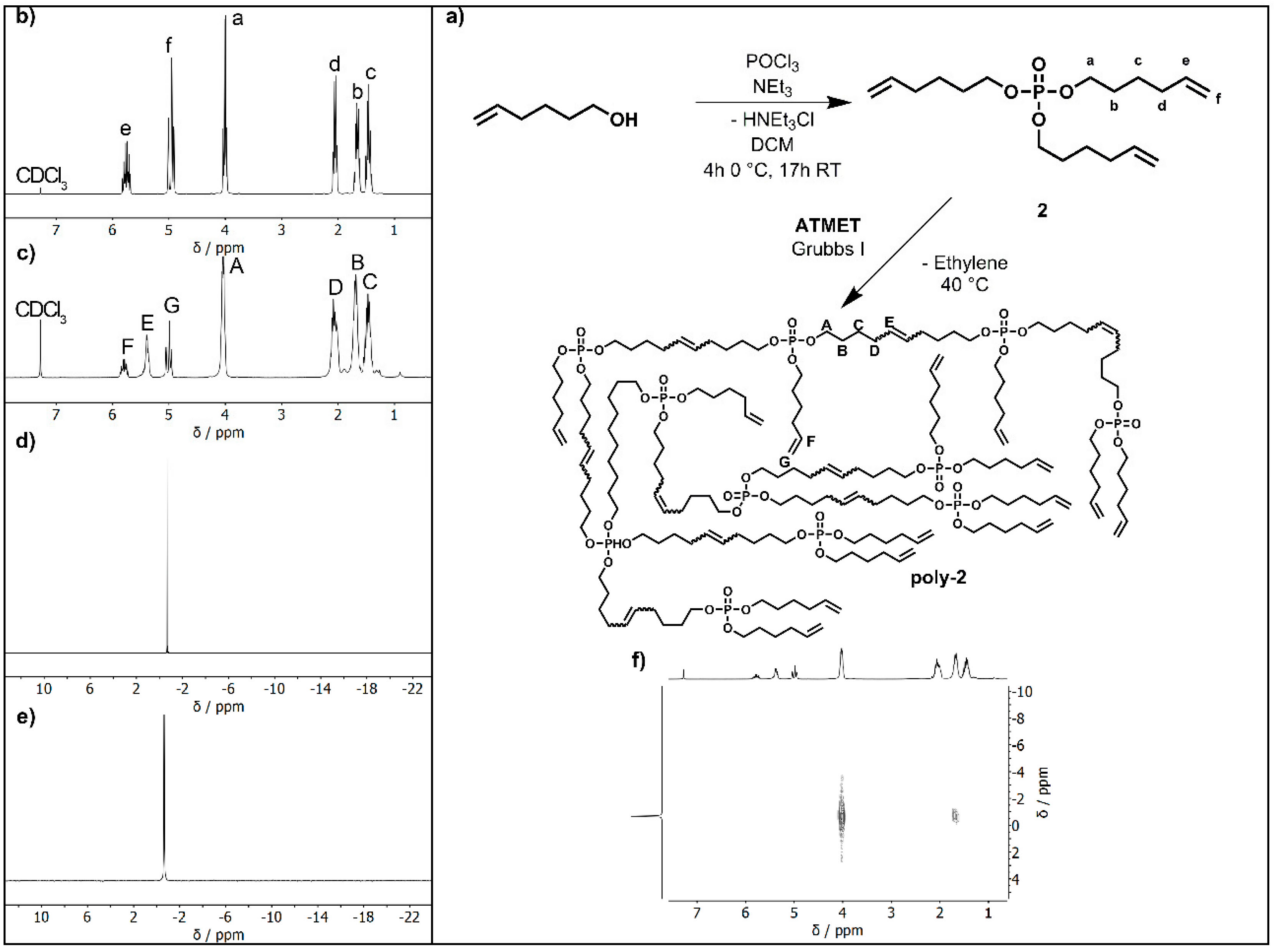
(**a**) Synthesis scheme of tri(hex-5-en-1-yl)phosphate (2) and poly(tri(hex-5-en-1-yl)phosphate) (poly-2); (**b**) ^1^H NMR spectra of 2; (**c**) ^1^H NMR spectra of poly-2; (**d**) ^31^P NMR spectra of 2; (**e**) ^31^P NMR spectra of poly-2; (**f**) ^1^H^31^P HMBC spectra of poly-2.

In addition to the aromatic poly-1, we also synthesized the aliphatic analogue from tri(hex-5-en-1-yl)phosphate (2) by ATMET polymerization (cf. Figure 2a and Experimental Part). Grubbs 1st generation catalyst was used for the polymerization which was terminated after ca. 15 min by the addition of ethyl vinyl ether, before cross-linking occurred and precipitated into hexane. The reaction was performed at 1 g and several polymer batches were combined to conduct the flame retardancy tests (after combining the different batches, SEC of the mixture showed a molar mass of ca. *M*_n_ 4400 g mol^−1^ (Figure S24)).

The successful polymerization of 2 was followed by ^1^H NMR spectroscopy. The signals of the terminal double bonds at 5.81 ppm and 5.15 ppm decreased, whereas an additional signal for the internal double bonds at 5.40 ppm was detected (Figure 2b,c). No shift in the phosphorus signal was observed, as the distance between the reactive olefins and the phosphorus is separated by the long alkyl chain (Figure 2d,e). The increase in thermal stability of poly-2 compared to 2 was marginal (*T*_on, 10%; poly-2_ = 206 °C and *T*_on, 10%; poly-2_ 215 °C).

### 2.1. Thermal Characterization of FRs

The analysis of both polymers by differential scanning calorimetry (DSC) revealed a large difference in the glass transition temperatures (*T*_g_): poly-2 with its long and flexible aliphatic chains between the phosphate groups exhibited a low *T*_g_ of −66 °C. In contrast, poly-1 exhibited a *T*_g_ which was 126 °C higher (*T*_g, poly-1_ = 60 °C) due to the rigid aromatic groups [31]. This difference in *T*_g_ also impacts the *T*_g_ of the final FR-containing epoxy resins and therefore must be considered. If a plasticizing-effect on the final FR epoxy resin is desired, the aliphatic FR may eliminate the need for additional plasticizer.

### 2.2. Pyrolysis: Thermal Decomposition via Thermogravimetric Analysis

**Figure 3:**
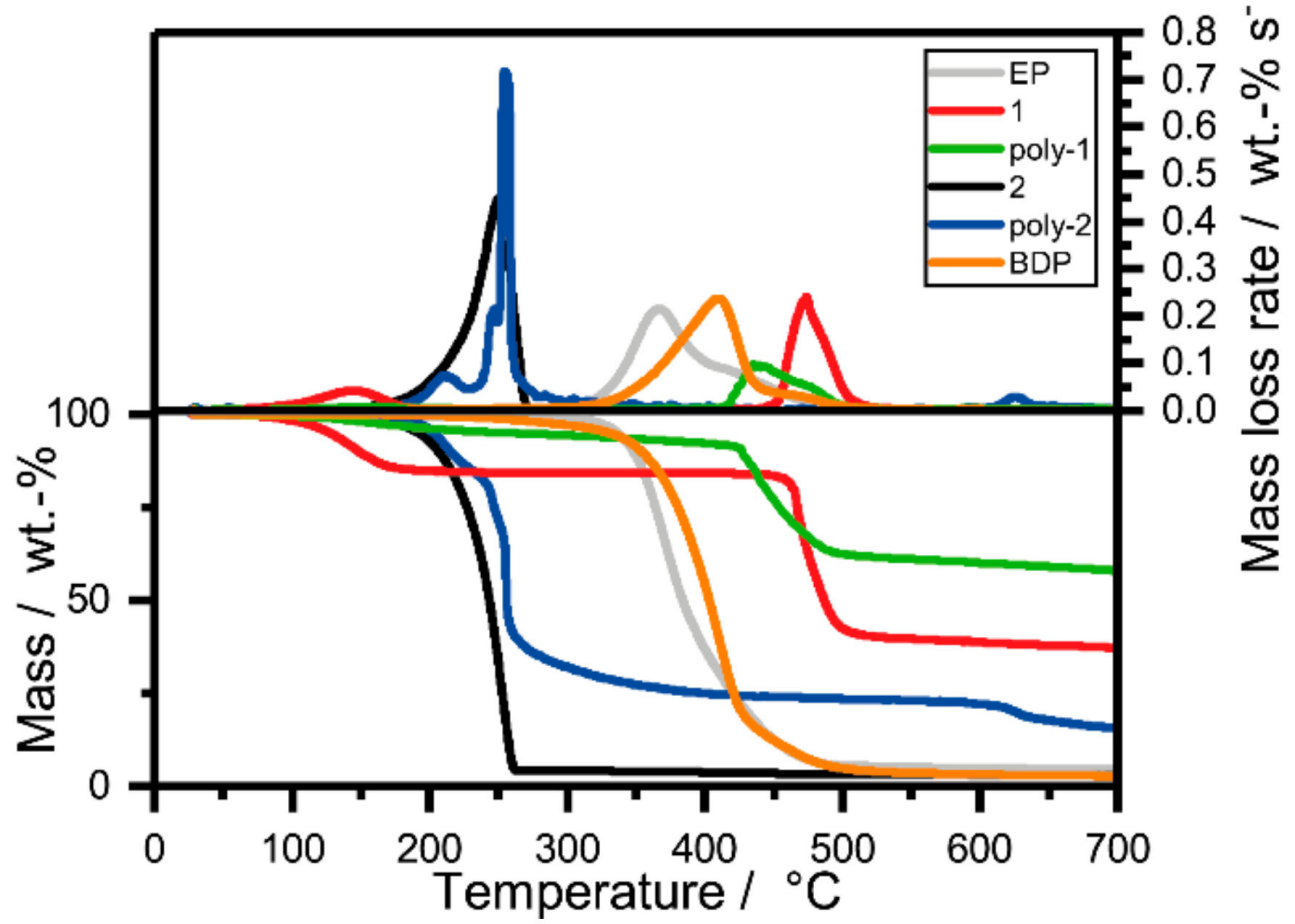
Top: Mass loss rate over temperature of pure flame retardants and the pure epoxy resin. Bottom: Pyrolysis investigations via thermogravimetric analysis (TGA; 10 K min^−1^; N_2_); increase of residue yield from aliphatic to aromatic and from monomeric to polymeric.

The decomposition of the FRs under pyrolytic conditions was investigated using thermogravimetric analysis (TGA) (Figure 3). During a fire, the burning is dominated by anaerobic pyrolysis producing volatile fuel, which is then combusted in the flame. This model is accurate for most polymeric materials in fire scenarios such as developing fires [32]. Therefore, it is also applicable for fire tests of polymeric materials like flaming combustion in the cone calorimeter. Moreover, the model applies to reaction to small flame tests such as limited oxygen index (LOI) and UL 94, where the extinguishment of a flame is monitored. TGA under nitrogen is the most common analytical method to investigate the pyrolysis controlling the burning of polymeric materials [32]. The mass loss curve of 1 has two distinct decomposition steps: the first mass loss step of 1 at *T*_dec_ = 143 °C is likely due to the cleavage of PO-Ar or P-OAr bonds. This behavior is indicated by the release of aromatic compounds which were identified by TGA coupled with FTIR spectroscopy (Figure S11). The resulting radicals of the bond cleavage initiate a radical polymerization reaction of the vinyl groups, resulting in a cross-linked polymer. This increase in molar mass prevents any further release into the gas phase. In contrast, poly-1 has a steady decrease in mass; it has only few free double bonds available which allow for further cross-linking. Consequently, end-groups are continually eliminated. The second decomposition step is the main decomposing step of the material, which is at the maximum degradation temperature *T*_max_ = 470 °C for 1 and at *T*_max_ = 435 °C for poly-1. The slight reduction in *T*_max_ for poly-1 is explained with the fact that poly-1 has only few vinyl groups for post cross-linking available; therefore, 1 is more efficient in its cross-linking reaction during the first mass loss step, resulting in a high molar mass, cross-linked network. The aliphatic compounds only experience one decomposition step, which is at much lower temperatures compared to the aromatic compounds. The difference between 2 and poly-2 is only marginal with 19 °C (*T*_max, 2_ = 250 °C, *T*_max, poly-2_ = 269 °C).

Considering the decomposition temperature of the epoxy resin (EP, based on bisphenol A diglycidylether (DGEBA) and 2,2′-dimethyl-4,4′-methylene-bis-(cyclohexylamine) (DMC), (*T*_max, epoxy_ = 366 °C)), another important observation was made: The aliphatic FRs decomposed at lower temperatures than the matrix, whereas the aromatic compounds decomposed at a higher temperatures than the matrix, resulting in potentially less interaction between matrix and FR (Figure 3). As a benchmark, the commercially available and industrially-used FR bisphenol-A diphenyl phosphate (BDP) was chosen, as it was already used successfully in epoxy resins [33,34]. BDP has a decomposition temperature which is similar to the one of the epoxy resin (*T*_max, BDP_ = 409 °C).

There is also a clear difference in residue amounts between the aliphatic and the aromatic materials: the residue increases from aliphatic to aromatic and from monomer to polymer. Residues increased from 3 wt.-% (2) to 37 wt.-% for 1 and from 24 wt.-% (poly-2) to 60 wt.-% for poly-1. The large difference in residues between aliphatic and aromatic compounds is explained by the interaction of phosphorus species with aromatic components, resulting in polyaromatic residue [35].

### 2.3. Pyrolysis: Evolved Gas Analysis via TGA-FTIR

During TGA, the evolved gases were analyzed via FTIR (TGA-FTIR), giving insight into the decomposition products and therefore the process as a whole (Figure S9–S12). 2 decomposed in a single step at 259 °C: its main decomposition products were 5-hexen-1-ol, 1,5-hexadiene, and a phosphate-species, as has been previously reported (Figure S9) [36]. poly-2 exhibited disparate products during decomposition: at 216 °C, the spectrum shares most similarities to 1,3-hexadiene (c,t), especially by the bands at 999 and 905 cm^−1^, but also those at 1806, 1605, 1460, 1316, and 1174 cm^−1^ (Figure S10) [37]. At 259 °C, 2-octene (c,t) and *trans*-1,4-hexadiene were identified, the former by those bands at 1460, 1405, and 692 cm^−1^, and the latter by the bands at 971 and 919 cm^−1^. The FTIR spectra of monounsaturated hydrocarbons share many similarities, and the spectra are likely caused by the overlap of several species. For 1, the spectrum at 143 °C revealed the production of aromatic products including *p*-Cresol, 4-ethylphenol, and *p*-tolyl acetate, the latter a product of rearrangement reactions, and identified by the bands at 1786 (C=O) [38,39], 1372, 1011, and 908 cm^−1^ (Figure S11). [37] At 470 °C, the spectrum revealed the presence of bisphenol A, characteristically seen in the decomposition of epoxy resins [40,41,42,43], providing evidence for cross-linking reactions occurring at elevated temperatures. For poly-1, two distinct decomposition products were identified at *T*_max_: at 447 °C, the main product was phenol, while at 487 °C, the production of benzene was clearly visible by the band at 672 cm^−1^ (Figure S12).

### 2.4. Thermal Characterization of FRs in Epoxy Resins

The FR-performance of the aliphatic and aromatic FRs was studied in an epoxy resin (EP) based on DGEBA and DMC. The epoxy plates were prepared by mixing DGEBA with DMC in the presence of 10 wt.-% of each FR, pouring the mixture into aluminum molds of desired dimensions, followed by curing for 3 h at 150 °C. The *T*_g_ of the epoxy resin was 155 °C. Typically, additive FRs act as plasticizers of the epoxy resin and reduce the glass transition temperature (*T*_g_) [44]. All flame-retarded epoxy resins with 10 wt.-% 1, poly-1, 2 and poly-2 exhibited lower *T*_g_s by 6–28 °C compared to the epoxy resin. 1 had almost no influence on the *T*_g_ of EP due to its ridged aromatic structure (*T*_g_ of EP-1: 149 °C), poly-1 reduced the *T*_g_ to 127 °C and EP-BDP lies in between with a *T*_g_ of 133 °C. In all cases, the addition of aliphatic FRs 2 and poly-2 resulted in a higher or equivalent decrease in *T*_g_ compared to the aromatic FRs (*T*_g, 2_ = 127 °C, *T*_g, poly-2_ = 149 °C). This difference in influence on the *T*_g_ was already expected due to the large difference in *T*_g_ of the pure FRs.

### 2.5. Pyrolysis: Evolved-Gas Analysis via TGA/TGA-FTIR

A crucial step towards understanding the FR mechanisms is analyzing the pyrolysis of the epoxy resins with FRs by TGA. The epoxy resin had a main decomposition step at 366 °C and therefore no overlap with the aliphatic or aromatic FRs (except the reference BDP). This behavior is not ideal, since it reduces the interaction between the matrix and FR. The mass loss curves of EP-2 and EP-poly-1 have two distinct signals. In the case of EP-2, the low molecular weight compound already boils from the matrix before the matrix decomposes. This is indicated by the mass loss of approx. 10 wt.-%, which is equal to the amount of FR in the epoxy resin. For EP-poly-1, the first mass loss step is explained with the loss of terminal groups. The temperatures of highest decomposition rate (*T*_max_) of EP-2 (*T*_max_ = 367 °C), EP-1 (*T*_max_ = 359 °C), EP-poly-1 (*T*_max_ = 361 °C) and EP-BDP (*T*_max_ = 357 °C) are very close to the *T*_max_ of the neat epoxy resin. However, poly-2 reduced *T*_max_ of EP to 334 °C. The broad range of decomposition is caused by the broad poly dispersity and the high number of low molecular weight fractions, which leave the matrix earlier compared to the higher molecular fragments. 

The residue at 700 °C increased for all tested FR containing EPs. EP-2 had a low increase to 5.1 wt.-% compared to 4.5 wt.-% of EP. EP-BDP had a residue of 8.2 wt.-%, which was notably higher. EP-1 had a residue of 9.1 wt.-%, while EP-poly-2 and EP-poly-1 were in a similar range with 13.3 wt.-% and 14.7 wt.-%, respectively, the latter presenting the greatest increase in residue yield.

The evolved-gas analysis of FR-containing resins during pyrolysis (Figure S13–S16) revealed the development of decomposition products unique to the individual FRs. All materials exhibited the spectrum of DGEBA-DMC at 371–375 °C except EP-poly-2, where this spectrum appeared at 349 °C. The EP-spectrum contained signals from Bisphenol A (e.g., 1603, 1510, 1255, 1176 cm^−1^) and ammonia (965, 930 cm^−1^). Only EP-2 contained a phosphate signal (1033 cm^−1^) [45] at 373 °C (Figure S13). Both EP-2 and EP-poly-2 exhibited gas production at *T* < *T*_max_ (266 and 287 °C, respectively): while for EP-2, the spectrum contained signals from 5-hexen-1-ol (3082, 2936, 1043, 918 cm^−1^) and a phosphate species (1033 cm^−1^) (Figure S13), the spectrum of EP-poly-2 displayed overlapped signals from 5-hexen-1-ol as well as longer-chained monounsaturated alcohols, as exemplified by the spectrum of oct-2-en-4-ol which contains the band at 967 cm^−1^ (Figure S14). These species resulted from the scission of the aliphatic chain between phosphate-moieties and then rearrangement reactions. For EP-1, the spectrum at 203 °C revealed the evolution of alkyl-substituted phenols such as *p*-*n*-propyl phenol, as identified by the bands at 1255, 1176 and 830 cm^−1^ which are prevalent throughout substituted aromatic molecules (Figure S15) [38,39]. Moreover, the spectrum at 437 °C displayed other substituted phenols that are unlike Bisphenol A, providing further support that 1 forms thermally stable polyaromatic compounds at elevated temperatures. EP-poly-1 also displayed similar polyaromatic molecules not stemming from Bisphenol A such as 4-(3-hydroxyisoamyl) phenol at 482 °C (Figure S16), as identified by the lack of signals at 1332, 747, and 686 cm^−1^ [37].

### 2.6. Pyrolysis: Condensed Phase Analysis via Hot-Stage FTIR

Investigations via hot-stage FTIR into the condensed phase activity of the FRs in EP revealed many similarities and some subtle differences between the individual FRs (Figure S17–S10). At 100 °C (Figure S17), the spectrum of EP is clearly visible in all materials, yet some additional bands are visible: for EP−2 and EP-poly-2, the band at 1025 cm^−1^ was pronounced and may correspond to (P-O) signals which are strong for the aliphatic FRs [45]. The bands at 1639 and 914 cm^−1^ were present in EP-2 and EP-1, as these corresponds to ν_s_(C=C) and δ_oop_(C-H) of the vinyl groups, respectively [37,38]. EP-poly-2, EP-1, and EP-poly-1 contained a distinctly strong band at 961 cm^−1^ from δ_oop_(C-H) of vinylene groups. The band at 734 cm^−1^ in EP-poly-1 may belong to δ_oop_(C-H) of *cis*-vinylene groups; these may show higher absorbance in aromatic systems [38,39]. At 300 °C (Figure S18), the spectrum of EP-poly-2 exhibited the most changes due to advanced decomposition (*T*_5%_ = 249 °C) compared to the other materials. Here, the band at 1083 cm^−1^ became pronounced; it may correspond to δ_as_(P-O-C) or ν(P=O) of Ar_2_-(P=O)-OH, especially because this band was prominent for EP-1 at 300 °C, but also all FR-containing spectra at 500 °C, thus implying the formation of aromatic P-compounds [38,39,45]. For EP-poly-2, the appearance of the strong band at 521 cm^−1^ may signify the evolution of monounsaturated hydrocarbons, as *cis*- and *trans*-alkenes as well as alkyl-substituted vinylenes exhibit strong signals from skeletal vibrations here. At 500 °C (Figure S19), the band at 1293 cm^−1^ appeared for all materials other than EP, and most prominently for EP-poly-2. This band may belong to ν(P=O) of (ArO)_3_-P=O, providing more evidence for the binding of aromatic rings by P-species of the FRs. Moreover, this band was clearly visible at 600 °C for all materials except EP, as the aromatic species are not stabilized by phosphorus. At 600 °C (Figure S20), many absorption bands were visible which were not present in EP, implicating a strong condensed phase mechanism of all tested FRs. Many of these bands have been characterized previously (Markwart et al., Battig et al.) [36,46]. Furthermore, the presence of several DGEBA-DMC typical bands (underlined in Figure S20) at 1593, 880, 823, 764, and 688 cm^−1^ suggest that the aromatic structures of DGEBA and its decomposition products were held intact through the formation of stable P-species.

### 2.7. Fire Behavior: Cone Calorimeter

**Figure 4:**
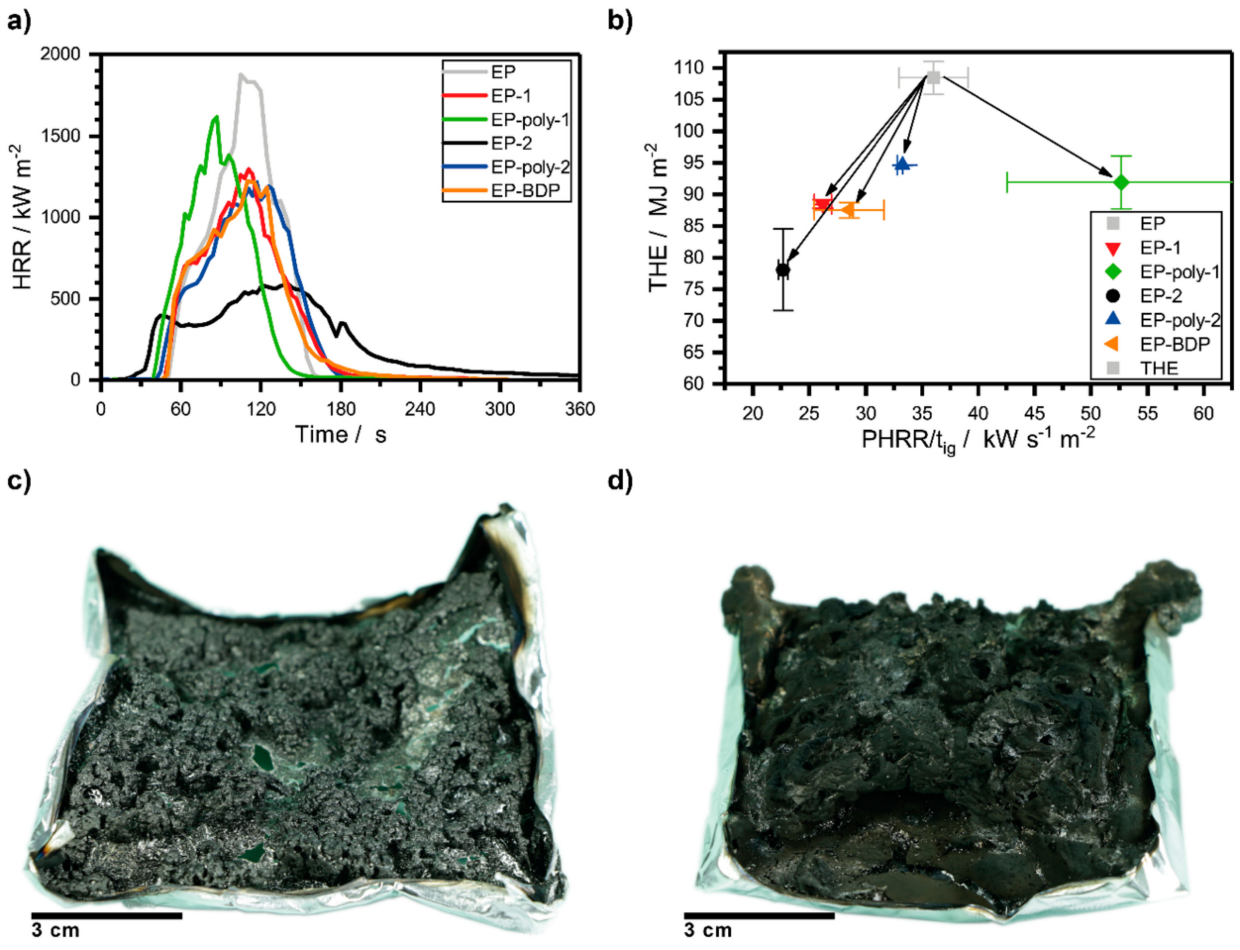
**(a**) Heat release rate (HRR) of epoxy resin and epoxy resin with flame retardants (FRs) measured by cone calorimeter. (**b**) Petrella plot of the different epoxy resins with all FRs having a positive effect (lowering total heat evolved (THE)), especially 2. (**c**) Residue of EP-poly-1 after cone calorimeter measurement, presenting a high graphitic residue yield but poor protective layer. (**d**) The residue of EP-poly-2 after cone calorimeter measurements, presenting a good protective layer by the production of voluminous, multicellular char.

Investigations with the cone calorimeter proved a significant effect of all FRs on epoxy resins (Figure 4b). The epoxy plates (100 × 100 × 4 mm^3^) were irradiated with a heat flux of 50 kW m^−2^, simulating a developing fire [47]. The results of the forced-flaming condition experiments underlined that the epoxy resin burned with a high heat release rate (HRR) and lost 99.3 wt.-% of its mass, presenting nearly no residue (Figure 4a and Table S4). The aliphatic FRs in resins exhibited a reduction of peak or heat release rate (PHRR), as well as reduction of fire growth rate (FIGRA = max. (HRR/*t*)). Especially 2 reduced the PHRR of EP significantly (885 vs. 1696 kW m^−2^) due to the formation of a voluminous char layer that insulated the underlying polymer. This behavior was clearly visible during the experiments as well as in the cross-sections of the residues, as the decomposition of the resin with 2 and the volatilization of its products acted as blowing agents, creating a voluminous intumescent char that shielded the underlying material from the heat source (Figure 4c,d). Moreover, it was very active in the gas phase, as evidenced by a reduction in the effective heat of combustion (EHC = total heat evolved/total mass loss), which is a parameter for gas phase activity [4]. The polymeric poly-2 did not exhibit the same type of FR efficacy due to a lower reactivity with the matrix. Notably, it only slightly lowered PHRR (1248 kW m^−2^) and was less active in the gas phase (EHC = 24.9 MJ kg^−1^). The aromatic FRs in resins did not exhibit a clear reduction in PHRR and FIGRA like 2. On the contrary, PHRR increased for EP-poly-1, and FIGRA was only slightly lowered. The epoxy resins loaded with 1 had a PHRR of 1194 kW m^−2^ and a FIGRA of 11.2 kW m^−2^ s^−1^ (pure epoxy: 1696 kW m^−2^ (PHRR) 15.5 kW m^−2^ s^−1^ (FIGRA)). Both values are on par to those of EP with the commercial FR BDP. Moreover, EP-1 exhibited a moderate 13% reduction of EHC (23.3 MJ kg^−1^) EP-poly-1 showed a PHRR of 1969 kW m^−2^ and a FIGRA of 15.0 kW m^−2^ s^−1^. The high PHRR is caused by the high decomposition temperature of the FR compared to the matrix, as seen in TGA measurements (Figure 3). All flame-retarded resins exhibited an increase in residue yield and a lowering of the total heat evolved (THE = total heat released (THR) at flame out) (Figure S22 and Table S4). The THE for the epoxy resins containing hb polymers were all comparable to EP-BDP (87.5 MJ m^−2^- 94.6 MJ m^−2^). The THE of EP-1 (88.4 MJ m^−2^) was comparable to that of EP-2 (78.1 MJ m^−2^). The epoxy resin loaded with 2 demonstrated the lowest PHRR (885 kW m^−2^, reduced by 48%) and THE (78.1 MJ m^−2^, reduced by 28%) and displayed a HRR curve corresponding to a charring material with a protective layer (Figure 4 a). For EP-poly-2 and EP-1, the HRR curves were nearly identical to EP-BDP: The shape of the HRR curves suggest a quasi-static HRR above ca. 60 s after ignition, indicating the formation of an insulating char layer. However, the insulating properties were soon overcome and additional fuel was transported into the flame, coming to a head shortly thereafter at PHRR. The HRR curve of EP-poly-1 illustrates that the sample ignited earlier than the pure EP, due to a reduced cross-linking density caused by the presence of FRs. Thereafter, fuel was continually fed into the flame before the FR could interact with EP, leading to a poor char layer production. For the polymers and especially for EP-poly-1, a lower reduction of PHRR and THE was detected, most probably due to the lower reactivity. The residue yields of epoxy resins loaded with 1 (5.3 ± 0.0 wt%) were higher than the neat epoxy (0.7 ± 0.1 wt%), yet much lower compared to the aliphatic monomeric phosphate (9.2 ± 0.1 wt.-%), most likely due to the high reactivity of 2. The resin with poly-1 had an increase in char residue (7.0 ± 1.5 wt.-%) compared to EP-1 (5.3 ± 0.0 wt.-%). Although the aromatic FRs were less effective in lowering PHRR and FIGRA in cone calorimeter measurements than the aliphatic counterparts, their efficacy in creating high residue yields in pyrolysis measurements should not go unnoted.

## 3. Materials and Methods

### 3.1. Materials

All chemicals were purchased from commercial suppliers as reagent grade and used without further purification. The monomer 2 was prepared according to literature [36].

### 3.2. Instrumentation and Characterization Techniques

#### 3.2.1. Size Exclusion Chromatography (SEC)

Size exclusion chromatography (SEC) measurements were performed in DMF at 60 °C with a PSS SecCurity system (Agilent Technologies 1260 Infinity, Santa Clara, CA, USA). Sample injection was performed by a 1260-ALS autosampler (Agilent) at 60 °C. GRAM columns (PSS) with dimensions of 300 × 80 mm, 10 μm particle size, and pore sizes of 100, 1000, and 10,000 Å were employed. The DRI Shodex RI-101 detector (ERC, Kawaguchi, Japan) and UV−vis 1260-VWD detector (Agilent) were used for detection. Calibration was achieved using PS standards provided by Polymer Standards Service. 

#### 3.2.2. Nuclear Magnetic Resonance (NMR)

Nuclear magnetic resonance (NMR) was performed in a Bruker Avance III 300 MHz and 500 MHz spectrometer (Billerica, MA, USA). All spectra were measured in either *d_6_*-DMSO or CDCl_3_ at 298 K. The spectra were calibrated against the solvent signal (CDCl_3_ (7.26 ppm) or *d_6_*-DMSO (2.50 ppm)) and analyzed using MestReNova 11 from Mestrelab Research S.L. (Santiago de Compostela, Spain)and Bruker Topspin 3.0 software (Billerica, MA, USA). 

#### 3.2.3. Electrospray Ionization Mass Spectrometry (ESI-MS)

Electron spray ionization mass (ESI-MS) was performed by A Q-Tof Ultima 3 from Waters Micromass Milford Massachusetts spectrometer (Milford, MA, USA). 1 mg of the sample was dissolved in 1 mL of solvent (THF or DCM) and injected into ionization chamber at 120 °C which operated with a stream of 100 L h^−1^ and a reference voltage of 35 V.

#### 3.2.4. Differential Scanning Calorimetry (DSC)

Differential scanning calorimetry (DSC) measurements were performed using a Mettler Toledo instrument (Columbus, OH, USA) 1/700 or DSC823 under nitrogen atmosphere at a heating rate of 10 °C min^−1^ starting from −140 °C.

#### 3.2.5. TGA-FTIR

Both decomposition and evolved gases were investigated under pyrolytic and thermo-oxidative conditions via Fourier transform infrared (FTIR) spectroscopy coupled with thermogravimetric analysis. For epoxy resins with and without FRs, 10 mg of powder attained from cryomilling were used for measurements, while 5 mg samples were measured for pure FRs. Using a TG 209 F1 Iris (Netzsch Instruments, Selb, Germany), samples were heated at a rate of 10 K min^−1^ from 30 to 900 °C under a nitrogen or synthetic air (80:20) gas flow of 30 mL min^−1^. The evolved gases were analyzed using a Tensor27 infrared spectrometer (Bruker Optics, Ettlingen, Germany), which was coupled to the TGA via a transfer line heated to 270 °C.

#### 3.2.6. Hot Stage FTIR

The condensed phase activity was monitored using hot-stage FT-infrared spectroscopy using a Vertex70 FTIR spectrometer (Bruker Optics, Ettlingen, Germany), equipped with an FTIR600 Linkam hot-stage cell (Linkam Scientific Instruments Ltd., Chilworth, UK). The samples were pressed into a KBr plate, loaded into the Linkam cell, and heated at a rate of 10 K min^−1^ from 30 to 600 °C under a nitrogen gas flow of 300 mL min^−1^.

#### 3.2.7. Cone Calorimeter

All epoxy resin samples were subjected to bench-scale forced flaming combustion using a cone calorimeter (Fire Testing Technology Ltd., East Grinstead, UK) at a distance of 35 mm between specimen and cone heater and a heat flux of 50 kW m^−2^ and in accordance with ISO 5660. Specimens sized 100 × 100 × 4 mm^3^ were conditioned at 23 °C and 50% relative humidity for at least 48 h and then subjected to irradiation. 

### 3.3. Synthetic Procedures

#### 3.3.1. Synthesis of the 4-Vinylphenol

Preparation of 4-vinylphenol was realized according to the method of Ricks-Laskoski et al [48]. To a dried three-necked, 2 L round bottom flask fitted with a dropping funnel and an mechanical stirrer 4-acetoxystyrene (53.36 g, 0.33 mol, 1 eq.) and tetrahydrofuran (200 mL) were added under an argon atmosphere and ice-cooling. Ice-cooling was continued throughout the process. 5 M solution of aqueous sodium hydroxide (100 mL, 0.8 mol, 2.4 eq.) was added dropwise to this solution. The yellow solution was stirred for 2 h under ice-cooling until the reaction was completed as indicated by TLC (SiO_2_: *R*_f_ = 0.75, 30 vol.% ethyl acetate/hexane). Then 1.5 M hydrochloric acid (340 mL) was added slowly to the crude reaction mixture until a pH of 7–5 was reached. The product was extracted with cold ethyl acetate (5 × 50 mL), washed with distilled water (3 × 50 mL), dried over magnesium sulfate and filtered. The product was distilled at reduced pressure while cooling with an ice bath to yield a crystalline solid (30.9 g, 78%). The product was stored at −20 °C under argon atmosphere and light exclusion to suppress self-initiated polymerization.

^1^H NMR (300 MHz, 298 K, DMSO-*d*_6_): δ/ppm = 9.53 (s, 1H), 7.28 (d, 2H, *J* = 9 Hz), 6.74 (d, 2H, *J* = 9 Hz), 6.61 (dd, 1H, *J* = 12 Hz/18 Hz), 5.68 (d, 1H, *J* = 18 Hz), 5.04 (d, 1H, *J* = 12 Hz).

^13^C{H} NMR (75.48 MHz, 298 K, DMSO-*d*_6_): δ/ppm = 157.3, 136.4, 128.2, 127.4, 115.3, 110.6.

#### 3.3.2. Synthesis of the Tris(p-vinylphenyl)phosphate (1)

To a dried three-necked, 1 L round bottom flask fitted with a dropping funnel and an mechanical stirrer, 4-vinylphenol (15 g, 0.125 mol, 4 eq.) dissolved in THF (300 mL) and triethylamine (12.63 g, 0.125 mol, 4 eq.) in THF (10 mL) at 0 °C and under an argon atmosphere. Then phosphoryl chloride (4.79 g, 0.031 mol, 1 eq.) in THF (30 mL) was added to the solution dropwise at 0 °C. Then the reaction was allowed to warm up to room temperature and stirred overnight at room temperature. The mixture was filtered and concentrated at reduced pressure while cooling in an ice-bath. The product was dissolved in DCM (100 mL) and extracted with water and a 1 M potassium hydroxide solution. The organic layer was dried over MgSO4, filtered and concentrated at reduced pressure yield a colorless viscous liquid (8.4 g, 67%). The product was stored at −20 °C under argon atmosphere and light exclusion to suppress self-initiated polymerization.

^1^H NMR (500 MHz, 298 K, CDCl_3_): δ/ppm = 7.29 (d, 2 H, *J* = 10 Hz), 7.11 (d, 2 H, *J* = 10 Hz), 6.58 (dd, 1 H, *J* = 10 Hz/15 Hz), 5.60 (d, 1 H, *J* = 15 Hz), 5.16 (d, 1 H, *J* = 10 Hz).

^13^C{H} NMR (75.48 MHz, 298 K, CDCl_3_): δ/ppm = 149.9 (d, *J* = 30 Hz), 135.6 (s), 135.2 (s), 127.6 (d, *J* = 6 Hz), 120.2 (d, *J* = 21 Hz), 114.3 (s).

^31^P{H} NMR (121.5 MHz, 298 K, CDCl_3_): δ/ppm = −17.72 (s). ESI MS: 405.14.

#### 3.3.3. ATMET Polymerization to Poly(1)

In a Schlenk tube fitted with a magnetic stirring bar, tris(p-vinylphenyl)phosphate (1) (1 g, 2.47 mmol) dissolved in 1-chloronaphthalene (1.67 g) was placed under an argon atmosphere at 40 °C. Then, the Grubbs-Hoveyda catalyst 2^nd^ gen. (4.65 mg, 0.007 mmol, 0.3 mol%) was added. The polymerization was carried out at a controlled vacuum of 5 × 10^−2^ mbar to remove the evolving ethylene. When the viscosity increased (typically after ca 5 min), the flask was filled with argon and the reaction was terminated by adding ethyl vinyl ether or methyl acrylate (5 mL). The brown solution was stirred for 1 h at 40 °C and then the mixture was concentrated at reduced pressure. The product was precipitated twice into hexane and dried at reduced pressure to give an off-white powder (yields 50–90%).

^1^H NMR (500 MHz, 298 K, CDCl_3_): δ/ppm = 7.57 (d, *J* = 16 Hz), 7.39 (m), 7.30 (d, *J* = 5 Hz), 7.16 (m), 6.92 (s), 6.59 (dd, *J* = 10 Hz/17 Hz), 5.61 (d, *J* = 17 Hz), 5.16 (d, *J* = 10 Hz).

^13^C{H} NMR (75.48 MHz, 298 K, CDCl_3_): δ/ppm = 149.8, 135.6, 134.8, 129.7, 127.9, 120.4, 118.3, 114.4.

^31^P{H} NMR (202.46 MHz, 298 K, CDCl_3_): δ/ppm = −17.73.

#### 3.3.4. Tri(hex-5-en-1-yl)phosphate (2)

Tri(hex-5-en-1-yl)phosphate was synthesized as described elsewhere [36].

#### 3.3.5. Poly(tri(hex-5-en-1-yl)phosphate) (poly-2) by ATMET Polymerization

In a Schlenk tube fitted with a magnetic stirring bar, tri(hex-5-en-1-yl)phosphate (2) (2 g, 5.81 mmol) was placed under an argon atmosphere at 60 °C. Then, Grubbs catalyst 1^st^ gen. (33.45 mg, 40.65 µmol, 0.7 mol%) was added. The polymerization was carried out at a controlled vacuum of 5 × 10^−2^ mbar to remove the evolving ethylene. When the viscosity increased, the flask was filled with argon and the reaction was terminated by adding ethyl vinyl ether (0.4 mL). The product was precipitated twice into hexane and dried at reduced pressure to give a viscous off-white polymer (yields 85–96%.).

^1^H NMR (300 MHz, 298 K, CDCl_3_): δ/ppm = 5.81 (ddt, *J* = 16.9, 10.2, 6.6 Hz), 5.40 (m, *J* = 5.5, 5.1 Hz), 4.99 (t, *J* = 13.9 Hz), 4.03 (m, 6H), 2.08 (qd, *J* = 14.8, 12.0, 8.3 Hz, 6H), 1.68 (h, *J* = 6.9 Hz, 6H), 1.47 (dp, *J* = 15.1, 7.5 Hz, 6H).

^31^P{H} NMR (121.5 MHz, 298 K, CDCl_3_): δ/ppm = −0.66.

#### 3.3.6. Epoxy Resins

All epoxy resins were prepared using bisphenol A diglycidylether (DGEBA) (Araldite MY740, Bodo Möller Chemie GmbH, Offenbach am Main, Germany) as the epoxide agent and 2,2′-dimethyl-4,4′-methylene-bis-(cyclohexylamine) (DMC) (Sigma Aldrich Co. LLC/Merck KgaA, Darmstadt, Germany) as the amine hardener. The materials were mixed, poured into aluminum molds of desired dimensions, then hardened at 150 °C for 3h. The flame retarded epoxy resins were produced in the same manner, except 10 wt.-% of the mixture was replaced with the respective flame retardant.

## 4. Conclusions

The herein presented aromatic and aliphatic, hyperbranched, halogen-free polyphosphoesters (hbPPEs) were synthesized by olefin metathesis polymerization and investigated as a flame retardant (FR) in epoxy resins. The impact on the *T*_g_ of the matrix was different for all tested materials. However, in all cases, the addition of aliphatic FRs 2 and poly-2 resulted in a higher or equivalent decrease of *T*_g_ compared to the aromatic FRs.

Regarding their flame retardant properties, the aromatic FRs proved a significant increase in residue yield and thermal stability in pyrolysis investigations compared to their aliphatic counterparts: the aromatic moieties acted as char precursors, thereby retaining significant residue yields. Moreover, condensed phase analysis concluded the formation of P-species for all tested resins with FRs.

Investigations with the cone calorimetry proved a significant effect of all FRs on epoxy resins during a developing fire. The aliphatic FRs were more effective on the tested matrix due to their greater overlap in decomposition temperature and thus better matrix interaction. The greater interaction resulted in a stronger reduction of peak of heat release rate (PHRR) and fire growth rate (FIGRA = max. (HRR/*t*)) than those resins with aromatic FRs. Especially 2 reduced the PHRR of EP significantly due to the formation of a voluminous char layer that insulated the underlying polymer. Moreover, 2 was very active in the gas phase, as evidenced by a reduction in the effective heat of combustion (EHC). While pyrolytic investigations proved high residue yields and interaction with the matrix for poly-2, it did not exhibit the same type of FR efficacy as 2 in cone calorimeter tests due to a less pronounced reactivity with the matrix in form of phosphorylation. The aromatic FRs were less effective in lowering PHRR and FIGRA in cone calorimeter measurements than the aliphatic counterparts, however, their efficacy in creating high residue yields in pyrolysis measurements should not go unnoted. The aromatic FRs might be more suited for materials with higher decomposition temperatures to increase the matrix-FR interaction. Moreover, the addition of a synergist may promote chemical interaction.

## Supplementary Materials

The following are available online: Table S1: Polymerization conditions of **1** with Grubbs Hoveyda 2^nd^ in 1-chlornaphthaline generation catalyst at 40 °C in solution., Figure S1: SEC curves (VWD-Signal 270 nm) of poly-1 in DMF polymerized with Grubbs 1st generation catalyst and Grubbs Hoveyda 2nd generation catalyst at 40 °C., Figure S2: ^1^H NMR (300 MHz in CDCl_3_ at 298 K) spectra of 4-vinylphenol., Figure S3: ^1^H-NMR (500 MHz in CDCl_3_ at 298 K) spectra of 1., Figure S4: ^31^P {H}-NMR (121 MHz in CDCl3 at 298 K) spectra of 1., Figure S5: ^1^H-MR (300 MHz in CDCl3 at 298 K) spectra of poly-1., Figure S6: ^31^P {H}-NMR (121 MHz in CDCl3 at 298 K) spectra poly-1., Figure S7: ^1^H-MR (300 MHz in CDCl3 at 298 K) spectra of poly-2., Figure S8: ^31^P {H}-NMR (121 MHz in CDCl3 at 298 K) spectra of poly-2., Figure S9: TGA-FTIR spectrum of **2** (top, black), identifying the main decomposition products (1,5-hexadiene; 5-hexen-1-ol and phosphate species, comparison shown in gray below) at specific decomposition temperature (259 °C) using references from NIST library., Figure S10: TGA-FTIR spectrum of **poly-2** (top, blue), identifying the main decomposition products (1,3-hexadiene (c,t) and 2-octene (c,t) and trans-1,4-hexadiene, comparison shown in gray below) at specific decomposition temperature (216 °C, 259 °C) using references from NIST library, Figure S11: TGA-FTIR spectrum of **1** (top, red), identifying the main decomposition products (p-tolyl acetate; p-cresol; 4-ethylphenol and bisphenol A, comparison shown in gray below) at specific decomposition temperature (143 °C, 470 °C) using references from NIST library., Figure S12: TGA-FTIR spectrum of poly-1 (top, green), identifying the main decomposition products (phenol and benzene, comparison shown in gray below) at specific decomposition temperature (447 °C and 487 °C) using references from NIST library., Figure S13: TGA-FTIR spectrum of EP-2 (top, black), identifying the main decomposition products (5-hexen-1-ol; phosphate species and decomposition products of the matrix, a comparison is shown in gray below) at specific decomposition temperature (266 °C, 373 °C) using references from NIST library., Figure S14: TGA-FTIR spectrum of EP-poly-2 (top, blue), identifying the main decomposition products (5-hexen-1-ol; oct-2-en-4ol and decomposition products of the matrix, a comparison is shown in gray below) at specific decomposition temperature (287 °C, 349 °C) using references from NIST library., Figure S15: TGA-FTIR spectrum of EP-1 (top, red), identifying the main decomposition products (p-n-propylphenol and decomposition products of the matrix, a comparison is shown in gray below) at specific decomposition temperature (203 °C, 371 °C, 437 °C) using references from NIST library., Figure S16: TGA-FTIR spectrum of EP-poly-1 (top, green), identifying the main decomposition products (4-(3-hydroxyisoamyl)phenol and decomposition products of the matrix, a comparison is shown in gray below) at specific decomposition temperature (375 °C, 482 °C) using references from NIST library., Figure S17: Results from hot-stage FTIR measurements, comparing the condensed phase spectra of EP-FRs at 100 °C., Figure S18: Results from hot-stage FTIR measurements, comparing the condensed phase spectra of EP-FRs at 300 °C., Figure S19: Results from hot-stage FTIR measurements, comparing the condensed phase spectra of EP-FRs at 500 °C., Figure S20: Results from hot-stage FTIR measurements, comparing the condensed phase spectra of EP-FRs at 600 °C, underlined bands are typical to DGEBA-DMC., Table S2: Glass transition temperatures (*T*_g_) of the flame retardant containing epoxy resins (measured by DSC), Figure S21: Mass loss (bottom) and mass loss rate (top) over *T* of neat epoxy resin and flame retardant containing epoxy resins from TGA measurements (10 K min^−1^; N_2_)., Table S3: TGA data of the flame retardant containing epoxy resins. T_5%_: Temperature at which 5% mass-loss happened; T_max_: Temperature of maximum degradation; Residue: Residue at 700 °C., Figure S22: Total heat released (THR) of epoxy resin and epoxy resin with flame retardant measured by cone calorimeter., Table S4: Results from cone calorimeter measurements of the flame retardant containing epoxy resins., Figure S23: Cross-linking of **1** at 300 °C in a silicon form for 2 h, producing a hard, cross-linked PPE resin., Figure S24: Chemical structure of Diglycidyl ether of Bisphenol A (DGEBA) and 2,2′-Dimethyl-4,4′-methylene-bis(cyclohexylamine) (DMC).
