# Supplementary material for: Aromatic vs. Aliphatic Hyperbranched Polyphosphoesters as Flame Retardants in Epoxy Resins
Source: Molecules. 2019 Oct 29;24(21):3901. doi: 10.3390/molecules24213901 (PMC6864611; doi:10.3390/molecules24213901)
Supplement: Supplementary file 1 [file molecules-24-03901-s001.pdf]

Supporting Information

for

Aromatic vs. aliphatic hyperbranched polyphosphoesters as flame retardants in epoxy resins

Jens C. Markwart‡[1,2], Alexander Battig‡[3], , Maria M. Velencoso[1], Dennis Pollok[1], Bernhard Schartel[3], Frederik R. Wurm*[1]*

[1] Max-Planck-Institut für Polymerforschung, Ackermannweg 10, 55128 Mainz, Germany; wurm@mpip-mainz.mpg.de
[2] Bundesanstalt für Materialforschung und -prüfung (BAM), Unter den Eichen 87, 12205 Berlin, Germany; bernhard.schartel@bam.de
[3] Graduate School Materials Science in Mainz, Staudinger Weg 9, 55128 Mainz, Germany
‡ These authors contributed equally to this work.

Corresponding author:

*E-mail: wurm@mpip-mainz.mpg.de; bernhard.schartel@bam.de

Table S1. Polymerization conditions of **1** with Grubbs Hoveyda 2nd in 1-chlornaphthaline generation catalyst at 40 ºC in solution.

**1st addition**					
**Polymer**	**%mol Catalyst**	**Time (min)**	**Mn (g/mol)**	**Mw (g/mol)**	**D**
1.2	0.32	60	1300	1400	1.1
1.3[i]	0.65	60	1700	2400	1.4
1.5[ii]	0.65	45	-	-	-
1.15	0.81	120	3000	8700	2.9
**2nd addition (after sampling of 1st addition)**					
**Polymer**	**%mol Catalyst**	**Time (min)**	**Mn (g/mol)**	**Mw (g/mol)**	**D**
1.2	0.32	60	1756	2390	1.36
1.3	0.65	30	4185	56306	13.45
1.5	0.32	25	4875	55333	11.35

[i] in bulk. [ii] cross-linked.

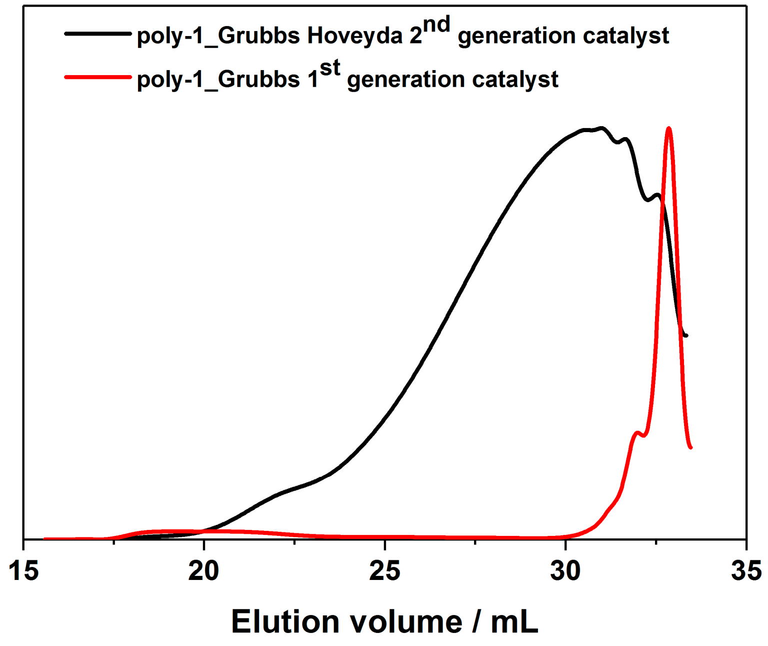

Figure S1. SEC curves (VWD-Signal 270 nm) of **poly-1** in DMF polymerized with Grubbs 1st generation catalyst and Grubbs Hoveyda 2nd generation catalyst at 40 ºC.

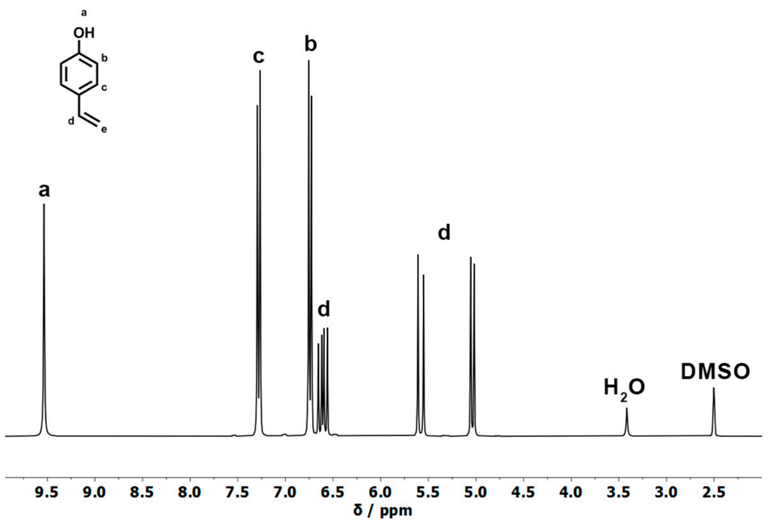

Figure S2. ^{1}H NMR (300 MHz in $CDCl_3$ at 298 K) spectra of 4-vinylphenol.

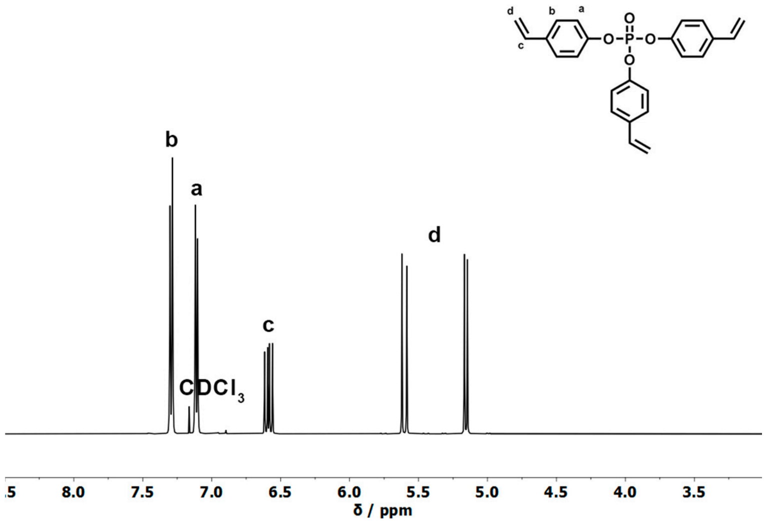

Figure S3. ^{1}H-NMR (500 MHz in $CDCl_3$ at 298 K) spectra of **1**.

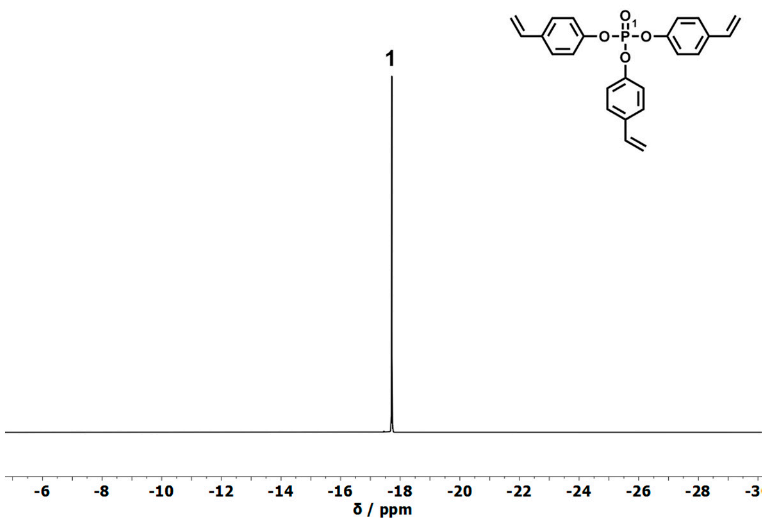

Figure S4. ^{31}P {H}-NMR (121 MHz in $CDCl_3$ at 298 K) spectra of **1**.

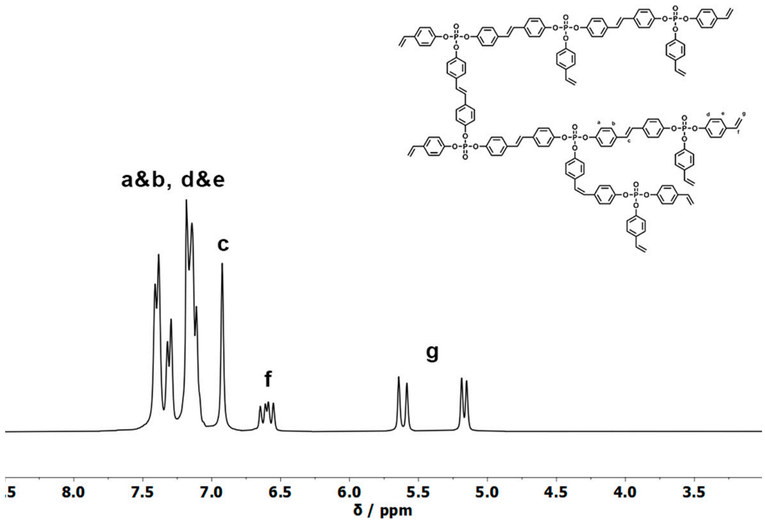

Figure S5. ^{1}H-MR (300 MHz in $CDCl_3$ at 298 K) spectra of **poly-1**.

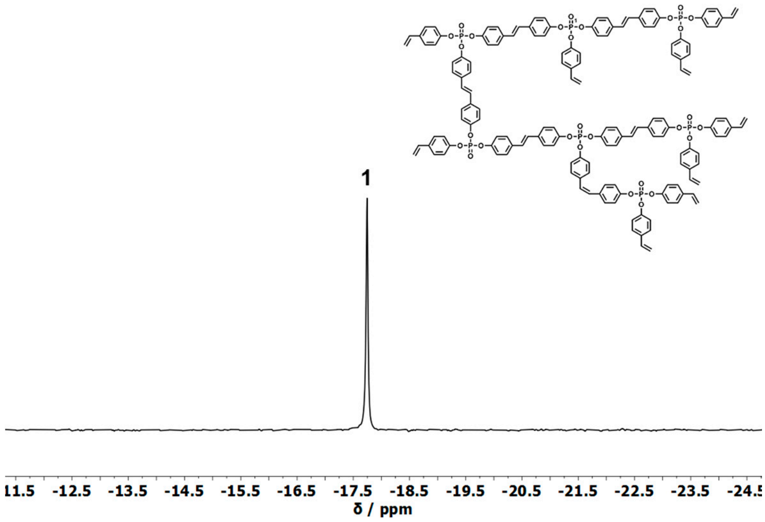

Figure S6. ^{31}P {H}-NMR (121 MHz in $CDCl_3$ at 298 K) spectra of **poly-1**.

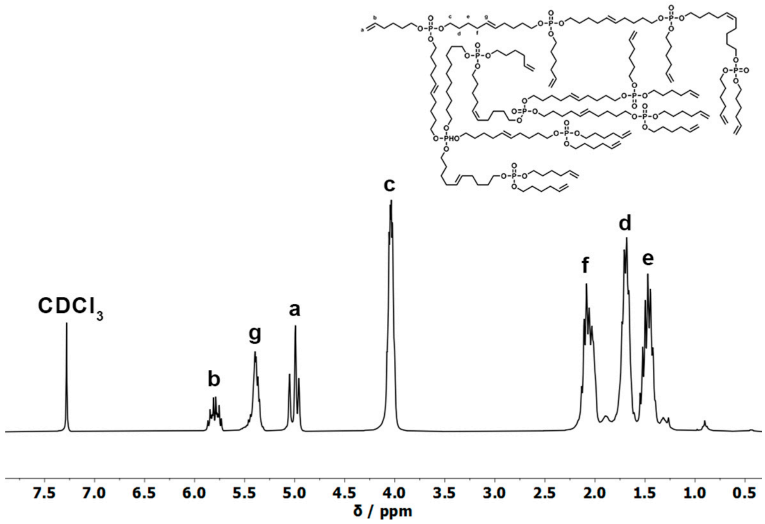

Figure S7. ^{1}H-MR (300 MHz in $CDCl_3$ at 298 K) spectra of **poly-2**.

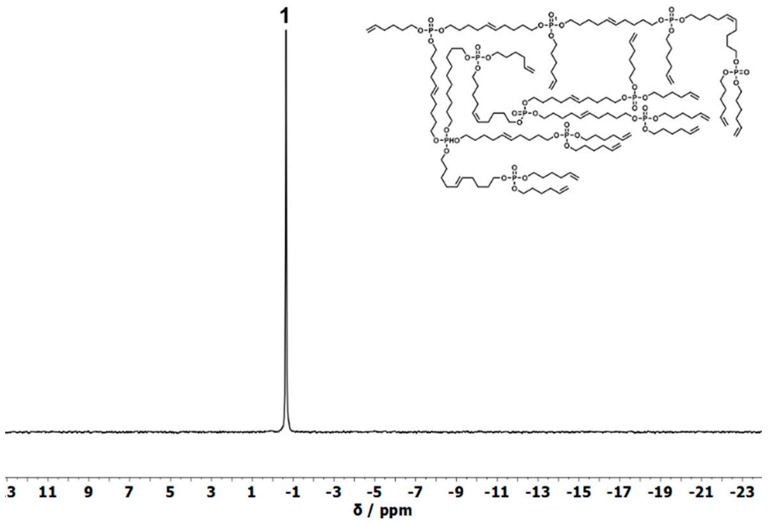

Figure S8. ^{31}P {H}-NMR (121 MHz in $CDCl_3$ at 298 K) spectra of **poly-2**.

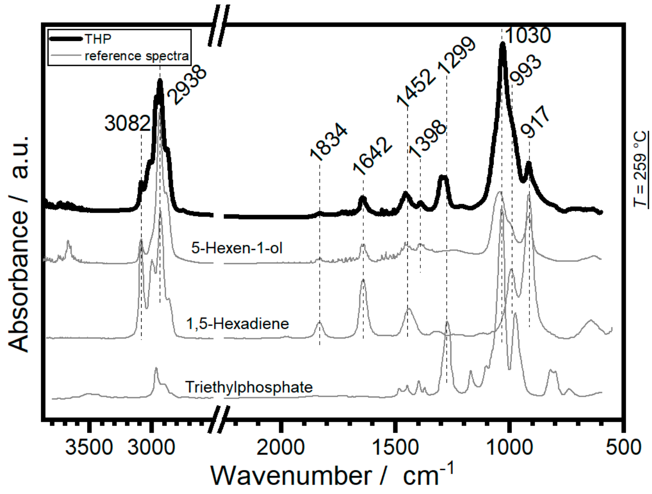

Figure S9. TGA-FTIR spectrum of **2** (top, black), identifying the main decomposition products (1,5-hexadiene; 5-hexen-1-ol and phosphate species, comparison shown in gray below) at specific decomposition temperature (259 °C) using references from NIST library.[1]

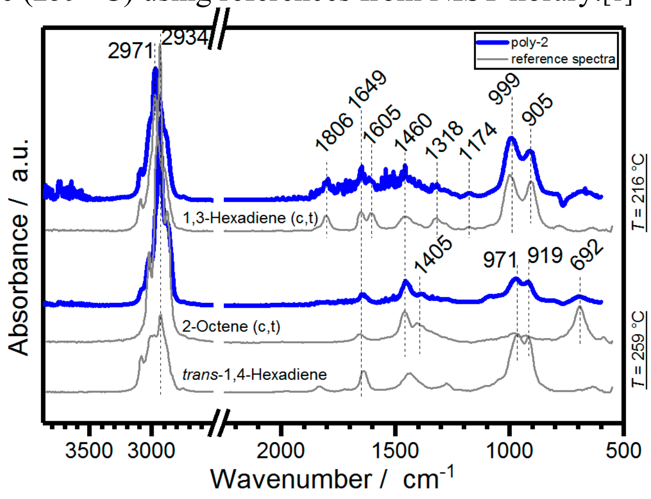

Figure S10. TGA-FTIR spectrum of **poly-2** (top, blue), identifying the main decomposition products (1,3-hexadiene (c,t) and 2-octene (c,t) and trans-1,4-hexadiene, comparison shown in gray below) at specific decomposition temperature (216 °C, 259 °C) using references from NIST library.[1]

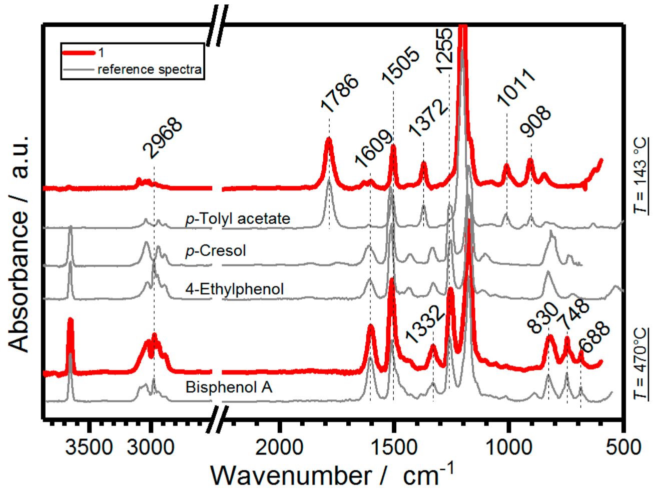

Figure S11. TGA-FTIR spectrum of **1** (top, red), identifying the main decomposition products (p-tolyl acetate; p-cresol; 4-ethylphenol and bisphenol A, comparison shown in gray below) at specific decomposition temperature (143 °C, 470 °C) using references from NIST library.[1]

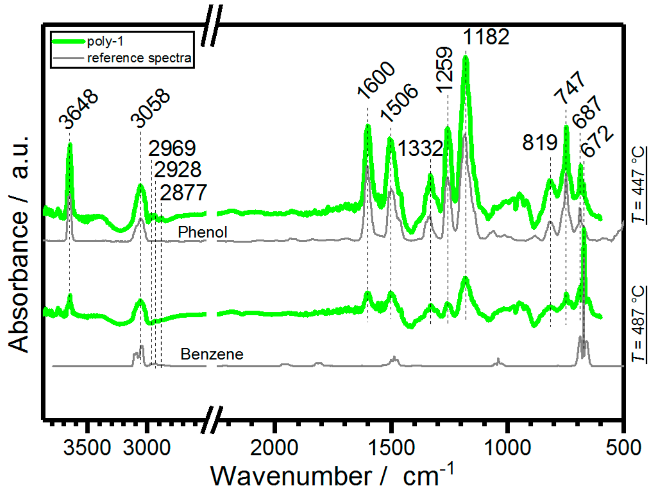

Figure S12. TGA-FTIR spectrum of **poly-1** (top, green), identifying the main decomposition products (phenol and benzene, comparison shown in gray below) at specific decomposition temperature (447 °C and 487 °C) using references from NIST library.[1]

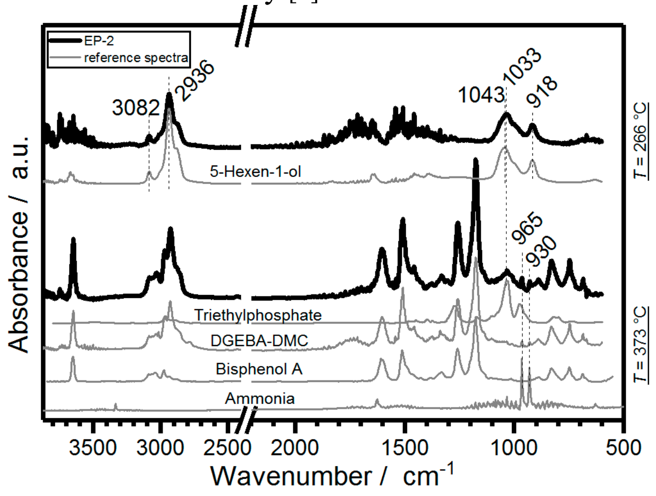

Figure S13. TGA-FTIR spectrum of **EP-2** (top, black), identifying the main decomposition products (5-hexen-1-ol; phosphate species and decomposition products of the matrix, a comparison is shown in gray below) at specific decomposition temperature (266 °C, 373 °C) using references from NIST library.[1]

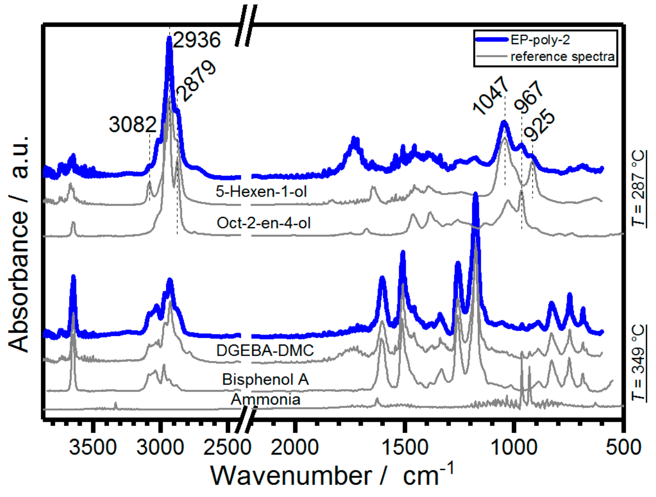

Figure S14. TGA-FTIR spectrum of **EP-poly-2** (top, blue), identifying the main decomposition products (5-hexen-1-ol; oct-2-en-4ol and decomposition products of the matrix, a comparison is shown in gray below) at specific decomposition temperature (287 °C, 349 °C) using references from NIST library.[1]

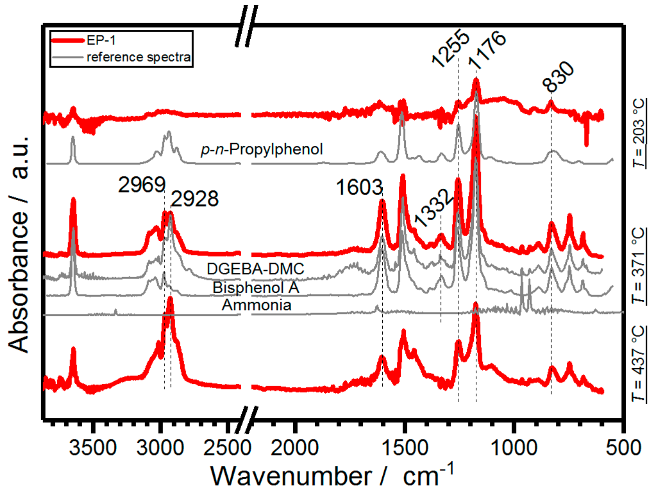

Figure S15. TGA-FTIR spectrum of **EP-1** (top, red), identifying the main decomposition products (p-n-propylphenol and decomposition products of the matrix, a comparison is shown in gray below) at specific decomposition temperature (203 °C, 371 °C, 437 °C) using references from NIST library.[1]

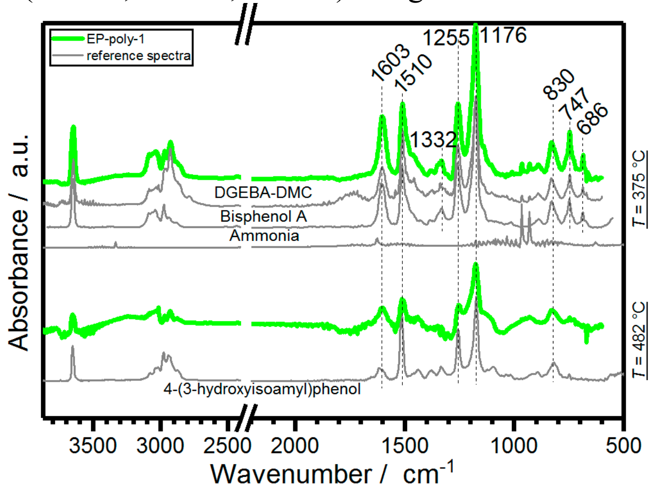

Figure S 16. TGA-FTIR spectrum of **EP-poly-1** (top, green), identifying the main decomposition products (4-(3-hydroxyisoamyl)phenol and decomposition products of the matrix, a comparison is shown in gray below) at specific decomposition temperature (375 °C, 482 °C) using references from NIST library.[1]

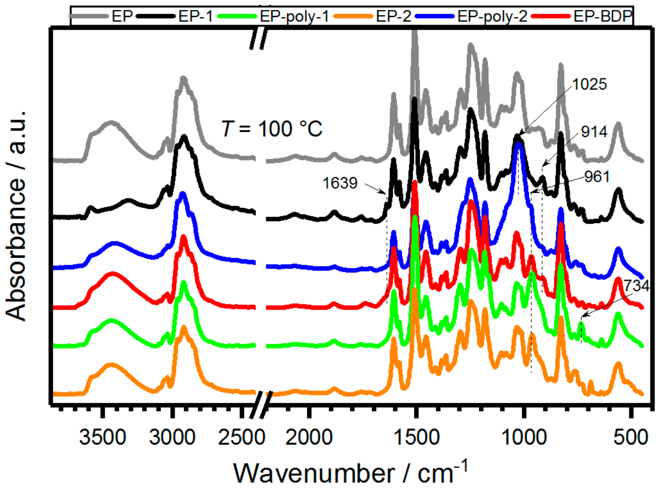

Figure S17. Results from hot-stage FTIR measurements, comparing the condensed phase spectra of EP-FRs at 100 °C.

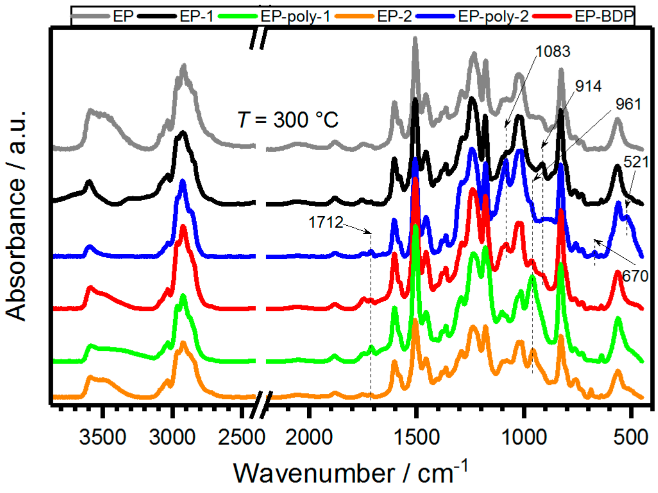

Figure S18. Results from hot-stage FTIR measurements, comparing the condensed phase spectra of EP-FRs at 300 °C.

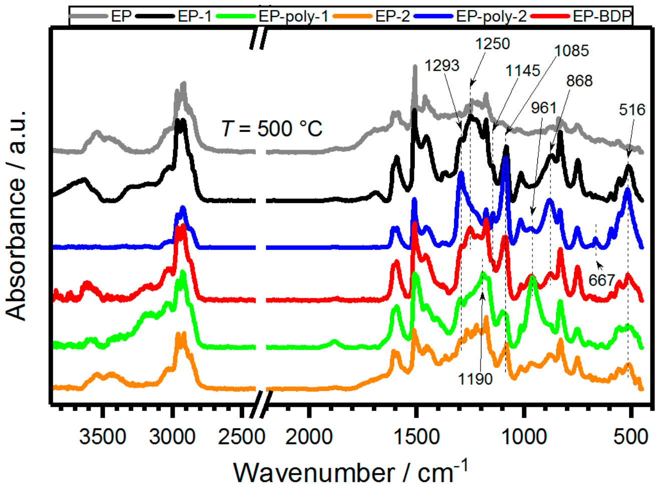

Figure S19. Results from hot-stage FTIR measurements, comparing the condensed phase spectra of EP-FRs at 500 °C.

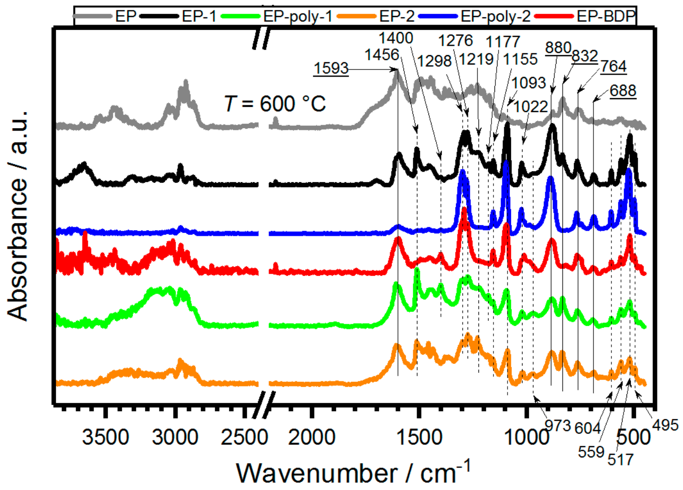

Figure S20. Results from hot-stage FTIR measurements, comparing the condensed phase spectra of **EP-FRs** at 600 °C, underlined bands are typical to DGEBA-DMC.

Table S2. Glass transition temperatures (T_g) of the flame retardant containing epoxy resins (measured by DSC).

**Material**	T_g
EP-1	149 ± 1
EP-poly-2	127 ± 3
EP- 2	113 ± 1
EP-poly-2	154 ± 2

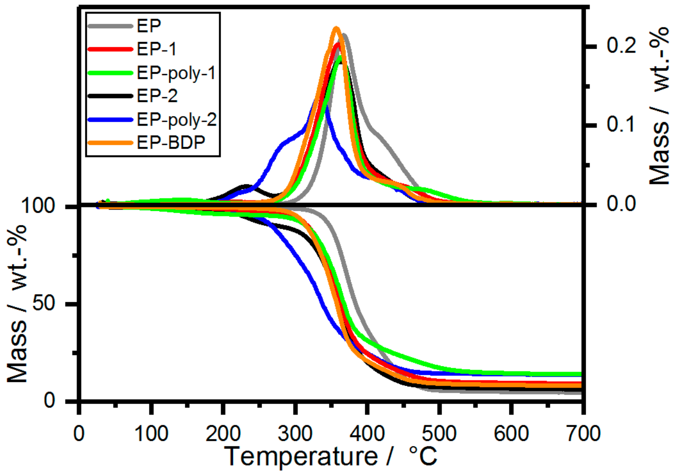

Figure S21. Mass loss (bottom) and mass loss rate (top) over T of neat epoxy resin and flame retardant containing epoxy resins from TGA measurements (10 K min^{-1}; N_2).

Table S3. TGA data of the flame retardant containing epoxy resins. $T_{5\%}$: Temperature at which 5% mass-loss happened; T_{max}: Temperature of maximum degradation; Residue: Residue at 700 °C.

Material	$T_{5\%}$ / °C	T_{max} / °C	Residue / wt.-%
DGEBA-DMC (EP)	338 ± 1	372 ± 1	4.5 ± 0.1
EP-1	279 ± 1	359 ± 1	9.1 ± 0.2
EP-poly-1	299 ± 3	361 ± 1	14.7 ± 0.5
EP-2	231 ± 1	367 ± 0	5.1 ± 0.1
EP-poly-2	249 ± 3	337 ± 2	13.3 ± 0.2
EP-BDP	304 ± 1	357 ± 0	8.2 ± 0.1

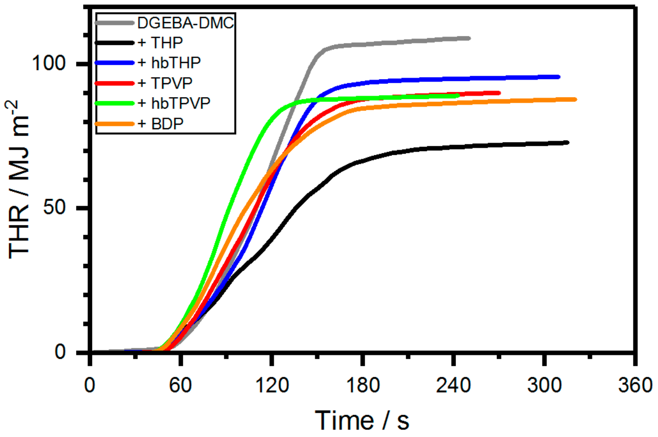

Figure S22. Total heat released (THR) of epoxy resin and epoxy resin with flame retardant measured by cone calorimeter.

Table S4. Results from cone calorimeter measurements of the flame retardant containing epoxy resins.

Material	THE / MJ m^{-2}	PHRR / kW m^{-2}	Residue / wt.-%	EHC / MJ kg^{-1}	FIGRA / kW m^{-2} s
DGEBA-DMC (EP)	108 ± 3	1696 ± 180	0.7 ± 0.1	26.9 ± 1.0	15.5 ± 2.3
EP-1	88 ± 1	1194 ± 100	5.3 ± 0.0	23.3 ± 0.2	11.2 ± 0.0
EP-poly-1	92 ± 4	1969 ± 353	7.0 ± 1.5	25.0 ± 1.5	15.0 ± 0.7
EP-2	78 ± 6	885 ± 16	9.2 ± 0.1	21.7 ± 1.8	9.0 ± 0.2
EP-poly-2	95 ± 0	1248 ± 32	5.1 ± 0.7	24.9 ± 0.2	9.0 ± 0.0
EP-BDP	87 ± 1	1180 ± 41	3.1 ± 0.2	22.7 ± 0.2	11.0 ± 0.7

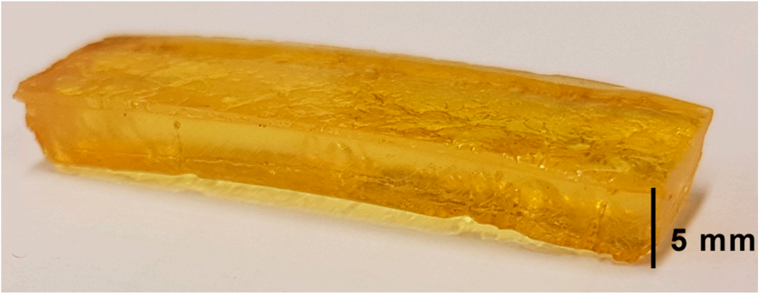

Figure S23. Cross-linking of **1** at 300 °C in a silicon form for 2 h, producing a hard, cross-linked PPE resin.

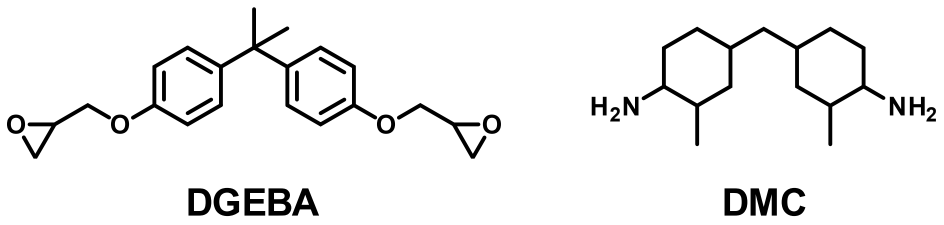

Figure S24. Chemical structure of Diglycidyl ether of Bisphenol A (DGEBA) and 2,2'-Dimethyl-4,4'-methylene-bis(cyclohexylamine) (DMC).

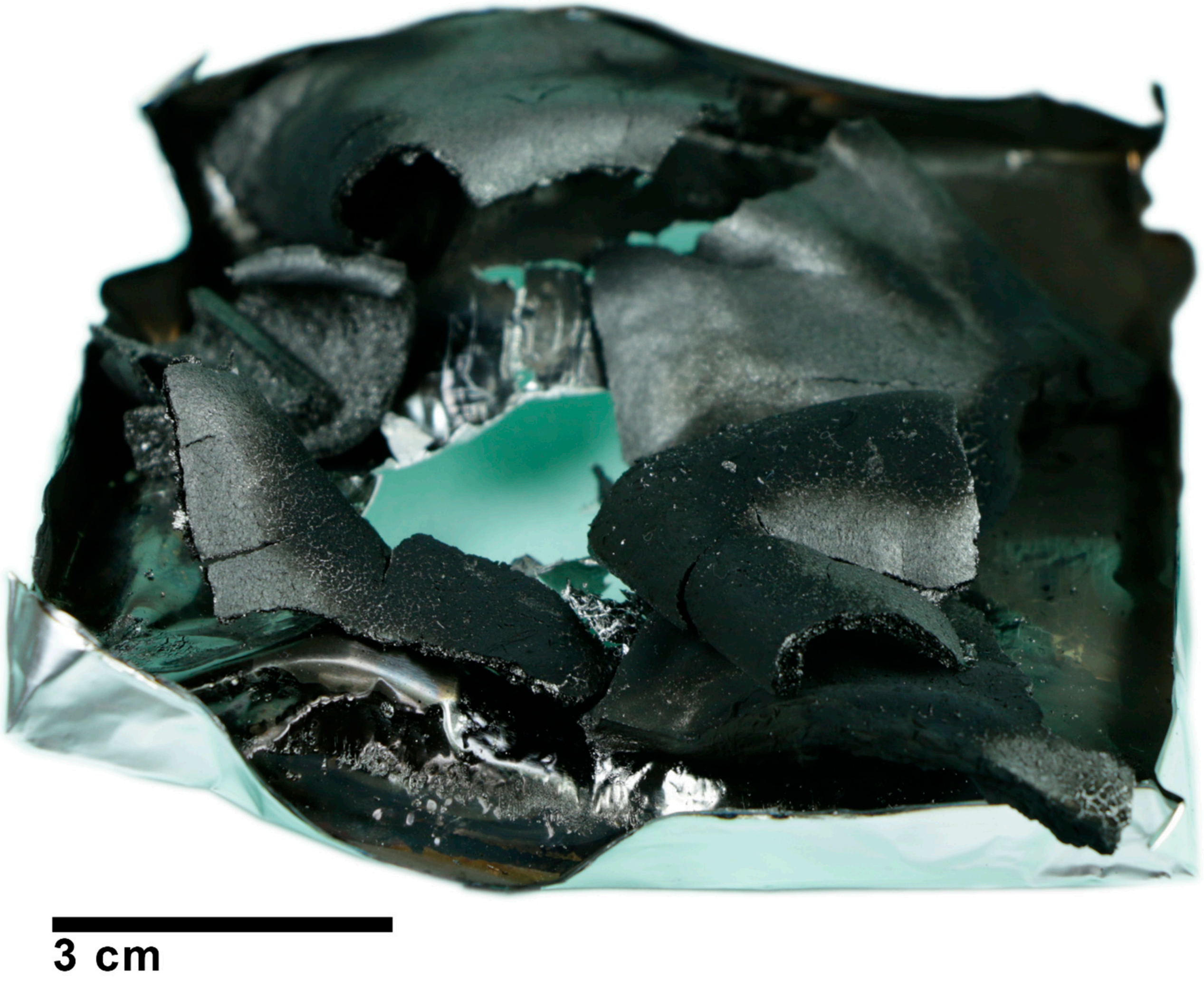

Figure S25. Residue of **EP-1** after cone calorimeter measurement, exhibiting a moderate protective layer effect from the formation of a rigid char layer.

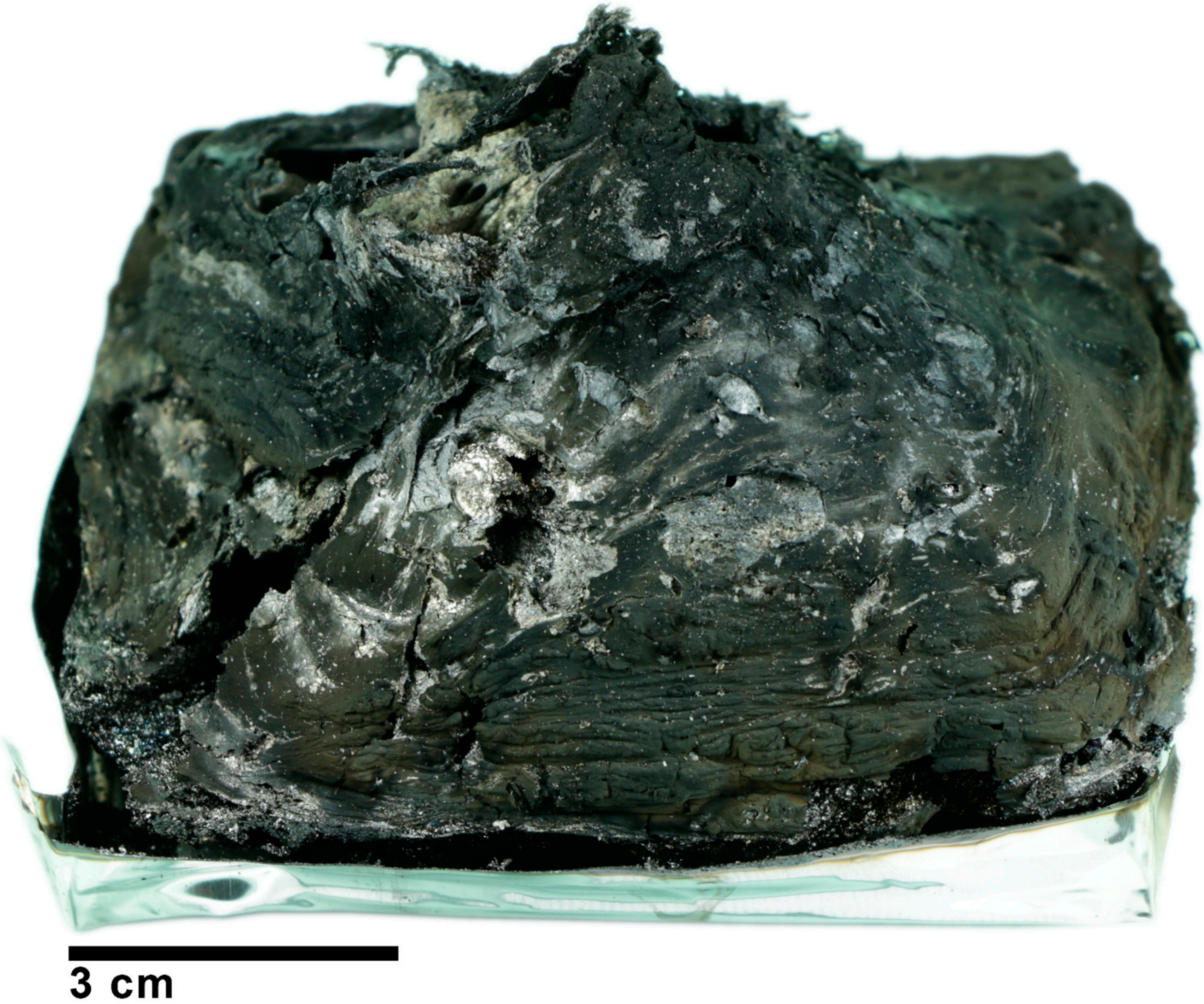

Figure S26. Residue of **EP-2** after cone calorimeter measurements, exhibiting a strong protective layer from the large voluminous, multicellular char.

References

1. Linstrom, P.J.; Mallard, W.G. NIST Chemistry WebBook, NIST Standard Reference Database Number 69. Available online: https://doi.org/10.18434/T4D303 (accessed on 27.08.2019).
